# Stochastic Entropy Solutions for Stochastic Scalar Balance Laws

**DOI:** 10.3390/e21121142

**Published:** 2019-11-22

**Authors:** Jinlong Wei, Bin Liu, Rongrong Tian, Liang Ding

**Affiliations:** 1 School of Statistics and Mathematics, Zhongnan University of Economics and Law, Wuhan 430073, China; weijinlong@zuel.edu.cn; 2 School of Mathematics and Statistics, Huazhong University of Science and Technology, Wuhan 430074, China; binliu@mail.hust.edu.cn; 3 College of Science, Wuhan University of Technology, Wuhan 430070, China; 4 School of Data Science and Information Engineering, Guizhou Minzu University, Guiyang 550025, China; ding2016liang@gzmu.edu.cn

**Keywords:** uniqueness, existence, stochastic entropy solution, stochastic kinetic formulation, Bhatnagar-Gross-Krook approximation, 60H15 (35L65 35R60)

## Abstract

We are concerned with the initial value problem for a multidimensional balance law with multiplicative stochastic perturbations of Brownian type. Using the stochastic kinetic formulation and the Bhatnagar-Gross-Krook approximation, we prove the uniqueness and existence of stochastic entropy solutions. Furthermore, as applications, we derive the uniqueness and existence of the stochastic entropy solution for stochastic Buckley-Leverett equations and generalized stochastic Burgers type equations.

## 1. Introduction

We are interested in the uniqueness and existence of the stochastic entropy solution for the following stochastic scalar balance law:

(1) $$d\rho \left(t,x\right)+{\mathrm{div}}_{x}\left(F\left(\rho \right)\right)dt+\sum_{i=1}^{d}\sum_{j=1}^{n}\partial_{x_{i}}B_{i,j}\left(t,\rho \right)\circ dW_{j}\left(t\right)=A\left(t,x,\rho \right)dt,\;x\in R^{d},\;t>0,$$

with a non-random initial condition:

(2) $${\rho \left(t,\cdot \right)|}_{t=0}=\rho_{0}\left(\cdot \right)\in L^{1}\left(R^{d}\right)\cap L^{\infty }\left(R^{d}\right).$$

Here ∘ is the Stratonovich convention and the use of the Stratonovich differential stems from the fact that ordinary differential equations with time dependent converging Brownian motion give rise stochastic differential equations of Stratonovich’s.

In (1), ρ(t,x) is a scalar random field. $W\left(t\right)={\left(W_{1}\left(t\right),W_{2}\left(t\right){,}_{\ldots },W_{n}\left(t\right)\right)}^{⊤}$ is an *n*-dimensional standard Wiener process on the classical Wiener space ($\Omega ,F,P,{\left(F_{t}\right)}_{t⩾0}$), i.e., Ω is the space of all continuous functions from [0,∞) to $R^{n}$ with locally uniform convergence topology, F is the Borel σ-field, P is the Wiener measure, ${\left(F_{t}\right)}_{t⩾0}$ is the natural filtration generated by the coordinate process W(t,ω)=ω(t). The flux function $F=\left(F_{1},F_{2}{,}_{\ldots },F_{d}\right):R\to R^{d}$ is assumed to be of class $W_{loc}^{1,1}$, i.e.,

(3) $$F\in W_{loc}^{1,1}\left(R;R^{d}\right).$$

The force *A* is supposed to satisfy that

(4) $$A\left(t,x,0\right)=0,A\in L_{loc}^{1}\left(\left[0,\infty \right);L^{1}\left(R^{d};W_{loc}^{1,1}\left(R\right)\right)+L^{\infty }\left(R^{d};W_{loc}^{1,1}\left(R\right)\right)\right).$$

For every 1⩽i⩽d,1⩽j⩽n, we assume

(5) $$B_{i,j}\in L_{loc}^{2}\left(\left[0,\infty \right);W_{loc}^{1,2}\left(R\right)\right).$$

When $B_{i,j}=0\;\left(1⩽i⩽d,1⩽j⩽n\right)$, (1) reduces to a deterministic partial differential equation known as the balance law

(6) $$\partial_{t}\rho \left(t,x\right)+{\mathrm{div}}_{x}\left(F\left(\rho \right)\right)=A\left(t,x,\rho \right),\;x\in R^{d},\;t>0.$$

The first pioneering result on the well-posedness of weak solutions for (6) is due to Kružkov [1]. Under the smoothness hypothesis on *F* and *A*, he obtained the existence in company with uniqueness of the admissible entropy solutions. For a completely satisfactory well-posedness theory for balance laws, one can consult to [2].

When A,F vanish and $\left(B_{i,j}\left(t,\rho \right)\right)=\mathrm{diag}\left(B_{1}\left(\rho \right),B_{2}\left(\rho \right){,}_{\ldots },B_{d}\left(\rho \right)\right)$, the equation has been discussed by Lions, Perthame and Souganidis [3,4]. Under the presumption that $B=\left(B_{1},B_{2}{,}_{\ldots },B_{d}\right)\in C^{2}\left(R\right)$, they developed a path-wise theory with quasi-linear (i.e., *B* is independent of the derivatives of ρ) multiplicative stochastic perturbations.

Recently there has been an interest in studying the effect of stochastic force on the corresponding deterministic equations, especially for the uniqueness and existence of solutions. Most of works are concentrated on the following form:

(7) $$d\rho \left(t,x\right)+{\mathrm{div}}_{x}\left(F\left(\rho \right)\right)dt=A\left(t,x,\rho \right)d\tilde{W}\left(t\right),\;x\in D,\;t>0,$$

where $\tilde{W}$ is a 1-dimensional Wiener process or a cylindrical Wiener process, $D\subset R^{d}$ is a bounded domain or $D=R^{d}$. When d=1, the bounded solution has been founded by Holden and Risebro [5], and Kim [6] for the forces A(ρ) and A(t,x), respectively, under assumptions that $\rho_{0}\in L^{\infty }$ and *A* has compact support. For general *A*, even the initial data is bounded, the solution is not bounded since the maximum principle is not available. Therefore, $L^{p}$ (1⩽p<∞) is a natural space on which the solutions are posed. When the force *A* is time independent, Feng and Nualart [7] developed a general theory for $L^{p}$-solutions (2⩽p<∞), but the existence was true only for d=1. Since then, Feng and Nualart’s result was generalized in different forms. For example, Bauzet, Vallet and Wittbold [8], Biswas and Majee [9] established the weak-in-time solutions, Karlsen and Storrøsten [10] derived the existence and uniqueness of stochastic entropy solutions for general d⩾1. At the same time, by using a different philosophy, Chen, Ding and Karlsen [11], Debussche and Vovelle [12], Hofmanová [13] also founded the well-posedness for $L^{p}$-solutions (1<p<∞) for any d⩾1. Furthermore, there are many other works devoted to discussing the Cauchy problem (7), (2), such as existence and uniqueness for solutions on bounded domains [14,15,16], existence of invariant measures [17,18] and long time behaviors [19] for solutions. For more details in this direction for random fluxes, we refer the readers to [20,21], and for more details for Lévy noises to see [22,23,24].

If we regard the last term in (7) as a multiplicative perturbation for the scalar conservation law:

$$\partial_{t}\rho \left(t,x\right)+{\mathrm{div}}_{x}\left(F\left(\rho \right)\right)=0,\;x\in D,\;t>0,$$

then the spatial average satisfies

$$\int_{D}\rho \left(t,x\right)dx=\int_{D}\rho_{0}\left(x\right)dx+\int_{0}^{t}\int_{D}A\left(s,x,\rho \left(s,x\right)\right)dxd\tilde{W}\left(s\right).$$

So the mass is not preserved in general. But if one considers the noise given in (1), then the mass is preserved exactly. It is one of our motivations to discuss the balance law

$$\partial_{t}\rho \left(t,x\right)+{\mathrm{div}}_{x}\left(F\left(\rho \right)\right)=A\left(t,x,\rho \right),\;x\in R^{d},\;t>0,$$

with the noise give by the form $\sum_{i=1}^{d}\sum_{j=1}^{n}\partial_{x_{i}}B_{i,j}\left(t,\rho \right)\circ dW_{j}\left(t\right)$. However, as far as we know the existing results for weak solutions to (1), (2) are few and all the results are concentrated on the following special case [25,26]:

$$d\rho \left(t,x\right)+b\left(t,x\right)\cdot \nabla_{x}\rho \left(t,x\right)dt+\sum_{i=1}^{d}\partial_{x_{i}}\rho \left(t,x\right)\circ dW_{i}\left(t\right)=0,\;x\in R^{d},\;t>0.$$

Further investigations are still needed. By using kinetic theory, we will prove the uniqueness and existence of the stochastic entropy solution to (1), (2). Here the stochastic weak solution and stochastic entropy solution are defined as follows:

**Definition** **1.** $\rho \in L_{loc}^{\infty }\left(\left[0,\infty \right);L^{\infty }\left(R^{d}\times \Omega \right)\right)\cap C\left(\left[0,\infty \right);L^{1}\left(R^{d}\times \Omega \right)\right)$ *is a stochastic weak solution of (1), (2), if for every* $\varphi \in D(R^{d})$ *,* $\int_{R^{d}}\rho \left(t,x\right)\varphi \left(x\right)dx$ *is an* $F_{t}$ *-semi-martingale and with probability one, the below identity*

∫Rdφ(x)ρ(t,x)dx−∫Rdφ(x)ρ0(x)dx−∫0t∫RdF(ρ)·∇xφ(x)dxds(8)=∑i=1d∑j=1n∫0t∘dWj(s)∫Rd∂xiφ(x)Bi,j(s,ρ)dx+∫0t∫RdA(s,x,ρ)φ(x)dxds

*holds true, for all* t∈[0,∞) *.*

**Remark** **1.** *Our motivation to define the weak solution comes from the classical theory of partial differential equations, i.e., ρ is a weak solution if it satisfies the equation in the sense of distributions: for every* $\psi \in D(\left[0,\infty \right)\times R^{d})$ *,*

$$\begin{array}{cc} & \int_{0}^{\infty }\int_{R^{d}}\partial_{t}\psi \left(t,x\right)\rho \left(t,x\right)dxdt+\int_{R^{d}}\rho_{0}\left(x\right)\psi \left(0,x\right)dx+\int_{0}^{\infty }\int_{R^{d}}F\left(\rho \right)\cdot \nabla_{x}\psi \left(t,x\right)dxdt\\ = & -\sum_{i=1}^{d}\sum_{j=1}^{n}\int_{0}^{\infty }\circ dW_{j}\left(t\right)\int_{R^{d}}B_{i,j}\left(t,\rho \right)\partial_{x_{i}}\psi \left(t,x\right)dx-\int_{0}^{\infty }\int_{R^{d}}A\left(t,x,\rho \right)\psi dxdt,\;P-a.s. \end{array}$$

*holds. Since ρ is continuous in time, the above identity is equivalent to (8).*

**Definition** **2.** *A stochastic weak solution of (1), (2) is a stochastic entropy solution, if for every* η∈Ξ *,*

(9) $$\partial_{t}\eta \left(\rho \right)+{\mathrm{div}}_{x}\left(Q\left(\rho \right)\right)+\sum_{i=1}^{d}\sum_{j=1}^{n}\partial_{x_{i}}Q_{i,j}\left(t,\rho \right)\circ {\dot{W}}_{j}\left(t\right)⩽h\left(t,x,\rho \right),\;P-a.s.,$$

*in the sense of distributions, i.e., for every* $\psi \in D_{+}\left(\left[0,\infty \right)\times R^{d}\right)$ *and almost all* ω∈Ω

$$\begin{array}{c} \int_{0}^{\infty }dt\int_{R^{d}}\partial_{t}\psi \left(t,x\right)\eta \left(\rho \right)dx+\int_{R^{d}}\psi \left(0,x\right)\eta \left(\rho_{0}\right)dx+\int_{0}^{\infty }\int_{R^{d}}Q\left(\rho \right)\cdot \nabla_{x}\psi \left(t,x\right)dxdt\\ +\sum_{i=1}^{d}\sum_{j=1}^{n}\int_{0}^{\infty }\circ dW_{j}\left(t\right)\int_{R^{d}}\partial_{x_{i}}\psi \left(t,x\right)Q_{i,j}\left(t,\rho \right)dx+\int_{0}^{\infty }\int_{R^{d}}h\left(t,x,\rho \right)\psi \left(t,x\right)dxdt⩾0, \end{array}$$

*where*

$$\begin{array}{c} Q\left(\rho \right)=\int^{\rho }\eta^{\prime }\left(v\right)F^{\prime }\left(v\right)dv,\;h\left(t,x,\rho \right)=A\left(t,x,\rho \right)\eta^{\prime }\left(\rho \right),\\ Q_{i,j}\left(t,\rho \right)=\int^{\rho }\eta^{\prime }\left(v\right)\partial_{v}B_{i,j}\left(t,v\right)dv,\;1⩽i⩽d,1⩽j⩽n, \end{array}$$

*and*

$$\Xi =\{c_{0}\rho +\sum_{k=1}^{n}c_{k}|\rho -\rho_{k}|,\;c_{0},\rho_{k},c_{k}\in R\;are\;constants\}.$$

**Remark** **2.** *We define the stochastic entropy solution by the inequality (9), and the source or motivation for this definition comes from the* ε→0 *limit of the following equation*

$$d\rho_{\varepsilon }\left(t,x\right)+{\mathrm{div}}_{x}\left(F\left(\rho_{\varepsilon }\right)\right)dt+\sum_{i=1}^{d}\sum_{j=1}^{n}\partial_{x_{i}}B_{i,j}\left(t,\rho_{\varepsilon }\right)\circ dW_{j}\left(t\right)-\varepsilon \Delta \rho_{\varepsilon }=A\left(t,x,\rho_{\varepsilon }\right)dt.$$

*Indeed, if one multiplies the above identity by* $\eta^{\prime }\left(\rho_{\varepsilon }\right)$ *, it yields that*

$$\partial_{t}\eta \left(\rho_{\varepsilon }\right)+{\mathrm{div}}_{x}Q\left(\rho_{\varepsilon }\right)+\sum_{i=1}^{d}\sum_{j=1}^{n}\partial_{x_{i}}Q_{i,j}\left(t,\rho_{\varepsilon }\right)\circ {\dot{W}}_{j}\left(t\right)-\varepsilon \eta^{\prime }\left(\rho_{\varepsilon }\right){\left(-\Delta_{x}\right)}^{\frac{\alpha }{2}}\rho_{\varepsilon }=h\left(t,x,\rho_{\varepsilon }\right).$$

*Since η is convex, with the help of the chain rule,*

$$\varepsilon \eta^{\prime }\left(\rho_{\varepsilon }\right)\Delta \rho_{\varepsilon }=\varepsilon \Delta \eta \left(\rho_{\varepsilon }\right)-\varepsilon \eta^{\prime \prime }\left(\rho_{\varepsilon }\right){\left|\nabla \rho_{\varepsilon }\right|}^{2}⩽\varepsilon \Delta \eta \left(\rho_{\varepsilon }\right).$$

*Therefore,*

$$\partial_{t}\eta \left(\rho_{\varepsilon }\right)+{\mathrm{div}}_{x}Q\left(\rho_{\varepsilon }\right)+\sum_{i=1}^{d}\sum_{j=1}^{n}\partial_{x_{i}}Q_{i,j}\left(t,\rho_{\varepsilon }\right)\circ {\dot{W}}_{j}\left(t\right)-\varepsilon {\left(-\Delta_{x}\right)}^{\frac{\alpha }{2}}\eta \left(\rho_{\varepsilon }\right)⩽h\left(t,x,\rho_{\varepsilon }\right).$$

*So the vanishing viscosity limit in the proceeding inequality leads to (9).*

We state our first main result on the Cauchy problem (1), (2).

**Theorem 1** (Stochastic kinetic formulation)**.** *Suppose that (3)–(5) hold.*

*(i) Let ρ be a stochastic entropy solution of (1), (2) and set* $u\left(t,x,v\right)=\chi_{\rho (t,x)}\left(v\right)=1_{\left(0,\rho (t,x)\right)}\left(v\right)-1_{\left(\rho (t,x),0\right)}\left(v\right)$ *. Then*

(10) $$u\in L_{loc}^{\infty }\left(\left[0,\infty \right);L^{\infty }\left(R^{d}\times \Omega ;L^{1}\left(R\right)\right)\right)\cap C\left(\left[0,\infty \right);L^{1}\left(R^{d+1}\times \Omega \right)\right),$$

*and it is a stochastic weak solution of the following linear stochastic transport equation (i.e., it is* $F_{t}$ *-adapted and satisfies the equation in the sense of distributions)*

(11) $$\partial_{t}u+f\left(v\right)\cdot \nabla_{x}u+\sum_{i=1}^{d}\partial_{x_{i}}u\circ {\dot{M}}_{i}\left(t,v\right)+A\left(t,x,v\right)\partial_{v}u=\partial_{v}m,\;\left(x,v\right)\in R^{d+1},\;t>0,$$

*supplied with*

(12) $${u\left(t,x,v\right)|}_{t=0}=\chi_{\rho_{0}\left(x\right)}\left(v\right),\;\left(x,v\right)\in R^{d+1}.$$

*Here* $f=F^{\prime }$ *,*

(13) $$M_{i}\left(t,v\right)=\sum_{j=1}^{n}\int_{0}^{t}\sigma_{i,j}\left(s,v\right)dW_{j}\left(s\right),\;\sigma_{i,j}\left(s,v\right)=\partial_{v}B_{i,j}\left(t,v\right),1⩽i⩽d,1⩽j⩽n.$$

$0⩽m\in L^{1}\left(\Omega ;D^{\prime }\left(\left[0,\infty \right)\times R^{d+1}\right)\right)$ *, satisfying, for every* T>0 *and for almost all* ω∈Ω *, m is bounded on* $\left[0,T\right]\times R^{d+1}$ *, supported in* $\left[0,T\right]\times R^{d}\times \left[-K,K\right]$ *(* $K={∥\rho ∥}_{L^{\infty }\left(\left(0,T\right)\times R^{d}\times \Omega \right)}$ *), and for every* $\phi \in D(R^{d+1})$ *,*

(14) $$\int_{0}^{t}\int_{R^{d+1}}\phi \left(x,v\right)m\left(ds,dx,dv\right),\;is\;F_{t}-adapted\;and\;continuous\;in\;t.$$

*(ii) Suppose that* $u\left(t,x,v\right)=\chi_{\rho (t,x)}\left(v\right)$ *. If* $u\in L_{loc}^{\infty }\left(\left[0,\infty \right);L^{\infty }\left(R^{d}\times \Omega ;L^{1}\left(R\right)\right)\right)\cap C(\left[0,\infty \right);$ $L^{1}\left(R^{d+1}\times \Omega \right))$ *is a stochastic weak solution of (11)–(14). We set* $\rho \left(t,x\right)=\int_{R}u\left(t,x,v\right)dv$ *, then*

(15) $$\rho \in L_{loc}^{\infty }\left(\left[0,\infty \right);L^{\infty }\left(R^{d}\times \Omega \right)\right)\cap C\left(\left[0,\infty \right);L^{1}\left(R^{d}\times \Omega \right)\right),$$

*and it is a stochastic entropy solution of (1), (2).*

**Remark** **3.** *(i) If u is a stochastic weak solution of (11)–(14), then (11) admits an equivalent representation: for every* $\phi \in D(R^{d+1})$ *, every* t∈[0,∞) *,* $\int_{R^{d+1}}\phi \left(x,v\right)u\left(t,x,v\right)dxdv$ *is* $F_{t}$ *-adapted and with probability one,*

$$\begin{array}{cc} & \int_{R^{d+1}}\phi \left(x,v\right)u\left(t,x,v\right)dxdv-\int_{0}^{t}\int_{R^{d+1}}f\left(v\right)\cdot \nabla_{x}\phi \left(x,v\right)dxdvds\\ = & \int_{R^{d+1}}\phi \left(x,v\right)u_{0}\left(x,v\right)dxdv+\sum_{i=1}^{d}\int_{0}^{t}\int_{R}M_{i}\left(\circ ds,dv\right)\int_{R^{d}}\partial_{x_{i}}\phi \left(x,v\right)u\left(s,x,v\right)dx\\ & +\int_{0}^{t}\int_{R^{d+1}}\partial_{v}\left[A\left(s,x,v\right)\phi \left(x,v\right)\right]u\left(s,x,v\right)dxdvds-\int_{0}^{t}\int_{R^{d+1}}\partial_{v}\phi \left(x,v\right)m\left(dx,dv,ds\right). \end{array}$$

*(ii) To the present case, we only study (1) with* F=F(ρ) *. However, if F depends on spatial variables, i.e.,* F=F(x,ρ) *, we can also establish a stochastic kinetic formulation up to a long and tedious calculations. In particular, for* $F\left(x,\rho \right)=b\left(x\right)F_{1}\left(\rho \right)$ *,* $B_{i,j}=0$ *and* A(t,x,ρ)dt *is replaced by* $A\left(\rho \right)dW_{t}$ *, we refer to [27], and for* $F\left(x,\rho \right)=b\left(x\right)F_{1}\left(\rho \right)$ *,* $B_{i,j}=\delta_{i,j}\rho$ *and* A(t,x,ρ)=0 *, to [28], and some related work, to [29].*

Our second result is on the uniqueness of the stochastic entropy solution.

**Theorem 2** (Uniqueness)**.** *Let* A(t,x,0)=0 *, that*

(16) $$A\in L_{loc}^{1}\left(\left[0,\infty \right);L^{1}\left(R^{d};W_{loc}^{1,1}\left(R\right)\right)+L^{\infty }\left(R^{d};W_{loc}^{1,\infty }\left(R\right)\right)\right),$$

(17) $${\left[\partial_{v}A\right]}_{+}\in L_{loc}^{1}\left(\left[0,\infty \right);L^{\infty }\left(R^{d};L_{loc}^{\infty }\left(R\right)\right)\right).$$

*Further, we assume that*

(18) $$F\in W_{loc}^{1,\infty }\left(R;R^{d}\right),\;B_{i,j}\in L_{loc}^{2}\left(\left[0,\infty \right);W_{loc}^{1,\infty }\left(R\right)\right)\;\left(1⩽i⩽d,1⩽j⩽n\right).$$

*Then there is at most one stochastic entropy solution ρ of (1), (2).*

As a corollary, we have

**Corollary 1** (Comparison Principle). *Let* $\rho_{1}$ *and* $\rho_{2}$ *be two stochastic entropy solutions of (1), with initial values* $\rho_{0,1}$ *and* $\rho_{0,2}$ *, if* $\rho_{0,1}⩽\rho_{0,2}$ *, then with probability one,* $\rho_{1}⩽\rho_{2}$ *.*

To make Theorem 2 more clear, we exhibit two representative examples here.

**Example** **1.** *The first example is concerned with the Buckley-Leverett equation (see [2]), which provides a simple model for the rectilinear flow of immiscible fluids (phases) through a porous medium. To be simple, nevertheless, to capture some of the qualitative features, we consider the case of two-phase flows (oil and water) in 1-dimensional space. In this issue, the Buckley-Leverett equation, with an external force, and a stochastic perturbation reads*

(19) $$\left\{ \begin{array}{c} d\rho \left(t,x\right)+\partial_{x}\left(F\left(\rho \right)\right)dt+\partial_{x}\rho \left(t,x\right)\circ dM(t)=\mu A(t,\rho )dt,\;x\in R,\;t>0,\\ {\rho \left(t,x\right)|}_{t=0}=\rho_{0}\left(x\right),\;x\in R, \end{array}\right.$$

*where* μ⩾0 *is a constant, W is a 1-dimensional standard Wiener process,* $\vartheta \in L_{loc}^{2}\left(\left[0,\infty \right)\right),\theta \in L_{loc}^{1}\left(\left[0,\infty \right)\right)$ *and*

(20) $$M\left(t\right)=\int_{0}^{t}\vartheta \left(s\right)dW\left(s\right),\;A\left(t,\rho \right)=\frac{\theta \left(t\right)\rho^{2}}{1+\rho^{2}}.$$

*The flux function F is determined using Darcy’s law and incompressibility of the two phases and is given by [30]:*

(21) $$F\left(\rho \right)=\frac{\sigma_{1}f_{1}\left(\rho \right)}{\sigma_{1}f_{1}\left(\rho \right)+\sigma_{2}f_{2}\left(\rho \right)}.$$

$\sigma_{1},\sigma_{2}>0$*denote the mobility of the oil and water phase, respectively, and*$f_{1}\left(\rho \right)$, $f_{2}\left(\rho \right)$*represent the relative permeability of oil and water, respectively.*$f_{1}$*and*$f_{2}$*are non-negative smooth functions and*$f_{1}+f_{2}>0$*.*

Applying Theorem 2, we obtain

**Corollary** **2.** *Assume that* $\rho_{0}\in L^{1}\left(R\right)\cap L^{\infty }\left(R\right)$ *. Then there exists at most one stochastic entropy solution ρ of (19). Moreover, if the initial data is non-negative, then the unique stochastic (if it exists) is non-negative as well.*

**Example** **2.** *The second example is concerned with a generalized Burgers equation (see [31]). This equation with a nonlinear stochastic perturbation of Brownian type, and a nonlinear nonhomogeneous term reads*

dρ(t,x)+divx(ζ|ρ(t,x)|αρ(t,x))dt(22)   +∑i=1d∂xi(ϑ(t)|ρ(t,x)|βρ(t,x))∘dWi(t)=λ(t)sin(x)ργdt, x∈Rd, t>0,

*associated with the initial value* $\rho_{0}$ *, where* $\zeta \in R^{d}$ *is a fixed vector,* α,β⩾0 *are constants,* 1⩽γ∈N *,* $\vartheta \in L_{loc}^{2}\left(\left[0,\infty \right)\right),\lambda \in L_{loc}^{1}\left(\left[0,\infty \right)\right).$ $W\left(t\right)=(W_{1}\left(t\right),W_{2}\left(t\right){,}_{\ldots },W_{d}\left(t\right))$ *is a d-dimensional standard Wiener process.*

From Theorem 2, we have

**Corollary** **3.** *Let* $\rho_{0}\in L^{1}\left(R\right)\cap L^{\infty }\left(R\right)$ *. If the stochastic entropy solutions of (22), (2) exists, then it is unique. In addition,* $\rho_{0}⩾0$ *implies the unique stochastic entropy solution (if it exists)* ρ⩾0 *.*

Our third result is on the existence of the stochastic entropy solution. And now we should assume the growth rates on the coefficients $B_{i,j}$, i.e., $B_{i,j}\left(t,\rho \right)$ is at most linear growth in ρ, and regularity property of *A* on spatial variables (e.g., Lipschitz continuous). In this case, we will establish the existence for stochastic entropy solutions. Up to a tedious calculation which is not technique, all calculations for $B_{i,j}\left(t,\rho \right)$ and A(t,x,ρ) are the same as $\rho \sigma_{i,j}\left(t\right)$ and A(t,ρ). To make our result present in a concise form, we only discuss the following stochastic balance law:

(23) $$\partial_{t}\rho \left(t,x\right)+{\mathrm{div}}_{x}\left(F\left(\rho \right)\right)+\sum_{i=1}^{d}\partial_{x_{i}}\rho \left(t,x\right)\circ {\dot{M}}_{i}\left(t\right)=A\left(t,\rho \right),\;x\in R^{d},\;t>0,$$

here $M_{i}\left(t\right)=\int_{0}^{t}\sigma_{i,j}\left(s\right)dW_{j}\left(s\right)$, (1⩽i,j⩽d).

**Theorem 3** (Existence)**.** *Let F, σ and A satisfy*

(24) $$F\in C^{1}\left(R;R^{d}\right),\;\sigma_{i,j}\in L_{loc}^{2}\left(\left[0,\infty \right)\right),\;A\in L_{loc}^{1}\left(\left[0,\infty \right);W^{1,\infty }\left(R\right)\right)\;and\;A\left(t,0\right)=0.$$

*Then there exists a stochastic entropy solution of the Cauchy problem (23), (2).*

If one argues Buckley-Leverett Equations (19)–(21) again, then by Theorems 2 and 3, we obtain

**Corollary** **4.** *Let F, ϑ and A be given in Example 1 and assume* $\rho_{0}\in L^{1}\left(R\right)\cap L^{\infty }\left(R\right)$ *. Then there exists a unique stochastic entropy solution ρ of (19). Moreover, if* $\rho_{0}⩾0$ *, then* ρ⩾0 *.*

The rest of the paper is structured as follows. In Section 2, we give some preliminaries. In Section 3 we present the proof of Theorem 1. The uniqueness and existence of stochastic entropy solutions are proved in Section 4 and Section 5. Section 4 is devoted to the proof of the uniqueness and in Section 5, we study the existence.

We end up the section by introducing some notations.

**Notations.**$D(R^{d})$, D(R), $D(\left[0,\infty \right)\times R^{d})$, $D(R^{d+1})$ and $D(\left[0,\infty \right)\times R^{d+1})$ stand for the sets of all smooth functions on $R^{d}$, R, $\left[0,\infty \right)\times R^{d}$, $R^{d+1}$ and $\left[0,\infty \right)\times R^{d+1}$ with compact supports, respectively. Correspondingly, $D_{+}\left(R^{d}\right)$, $D_{+}\left(R\right)$$D_{+}\left(\left[0,\infty \right)\times R^{d}\right)$, $D_{+}\left(R^{d+1}\right)$ and $D_{+}\left(\left[0,\infty \right)\times R^{d+1}\right)$ represent the non-negative elements in $D(R^{d})$, D(R), $D(\left[0,\infty \right)\times R^{d})$, $D(R^{d+1})$ and $D(\left[0,\infty \right)\times R^{d+1})$, respectively. ${\langle \;,\;\rangle }_{v}$ denotes the duality between D(R) and $D^{\prime }\left(R\right)$. ${\langle \;,\;\rangle }_{t,x,v}$ is the duality between $D(\left[0,\infty \right)\times R^{d+1})$ and $D^{\prime }\left(\left[0,\infty \right)\times R^{d+1}\right)$. C(T) denotes a positive constant depending only on *T*, whose value may change in different places. a.s. is the abbreviation of “almost surely”. The stochastic integration with a notation ∘ is interpreted in Stratonovich sense and the others is Itô’s. For a given measurable function *g*, $g_{+}$ is its positive portion, defined by $1_{g⩾0}g$, and $g_{-}={\left[-g\right]}_{+}$. $\mathrm{sgn}\left(g\right)=1_{g>0}-1_{g<0}$. N is natural numbers and d,n∈N. For notational simplicity, we set

$$a=(\begin{array}{cccc} a_{1,1}, & a_{1,2}, & \ldots , & a_{1,d}\\ a_{2,1}, & a_{2,2}, & \ldots , & a_{2,d}\\ \vdots & \vdots & \ddots & \vdots \\ a_{d,1}, & a_{d,2}, & \ldots , & a_{d,d} \end{array})=\frac{1}{2}(\begin{array}{cccc} \sigma_{1,1}, & \sigma_{1,2}, & \ldots , & \sigma_{1,n}\\ \sigma_{2,1}, & \sigma_{2,2}, & \ldots , & \sigma_{2,n}\\ \vdots & \vdots & \ddots & \vdots \\ \sigma_{d,1}, & \sigma_{d,2}, & \ldots , & \sigma_{d,n} \end{array})(\begin{array}{cccc} \sigma_{1,1}, & \sigma_{2,1}, & \ldots , & \sigma_{d,1}\\ \sigma_{1,2}, & \sigma_{2,2}, & \ldots , & \sigma_{d,2}\\ \vdots & \vdots & \ddots & \vdots \\ \sigma_{1,n}, & \sigma_{2,n}, & \ldots , & \sigma_{d,n} \end{array}).$$

## 2. Preliminaries

In this section, we give some useful lemmas that will serve us well later in proving our main results.

**Lemma** **1** (11). *has the following equivalent representation:*

(25) $$\partial_{t}u+f\left(v\right)\cdot \nabla_{x}u+\sum_{i=1}^{d}\partial_{x_{i}}u{\dot{M}}_{i}\left(t,v\right)-\sum_{i=1}^{d}\sum_{j=1}^{d}a_{i,j}\left(t,v\right)\partial_{x_{i},x_{j}}^{2}u+A\partial_{v}u=\partial_{v}m,\;\left(x,v\right)\in R^{d+1},\;t>0.$$

**Proof.**  Clearly, it suffices to show: for every $\phi \in D(R^{d+1})$, and for all t∈[0,∞),

$$\begin{array}{cc} & \sum_{i=1}^{d}\int_{0}^{t}\int_{R^{d+1}}\partial_{x_{i}}\phi \left(x,v\right)u\left(s,x,v\right)dxM_{i}\left(\circ ds,dv\right)\\ = & \sum_{i=1}^{d}\int_{0}^{t}\int_{R^{d+1}}\partial_{x_{i}}\phi \left(x,v\right)u\left(s,x,v\right)dxM_{i}\left(ds,dv\right)\\ & +\sum_{i=1}^{d}\sum_{j=1}^{d}\int_{0}^{t}ds\int_{R^{d+1}}a_{i,j}\left(s,v\right)\partial_{x_{i},x_{j}}^{2}\phi \left(x,v\right)u\left(s,x,v\right)dxdv. \end{array}$$

With the aid of stochastic Fubini’s theorem (see [32] Theorem 4.18), we have

$$\begin{array}{cc} & \sum_{i=1}^{d}\int_{0}^{t}\int_{R^{d+1}}\partial_{x_{i}}\phi \left(x,v\right)u\left(s,x,v\right)dxM_{i}\left(\circ ds,dv\right)\\ = & \sum_{i=1}^{d}\int_{0}^{t}\int_{R^{d+1}}\partial_{x_{i}}\phi \left(x,v\right)u\left(s,x,v\right)dxM_{i}\left(ds,dv\right)\\ & +\frac{1}{2}\sum_{i=1}^{d}\int_{R}{\left[\int_{R^{d}}\partial_{x_{i}}\phi \left(x,v\right)u\left(\cdot ,x,v\right)dx,M_{i}\left(\cdot ,v\right)\right]}_{t}dv, \end{array}$$

where ${\left[\cdot ,\cdot \right]}_{t}$ denotes the joint quadratic variation, thus it is sufficient to demonstrate

$$\begin{array}{cc} & \sum_{i=1}^{d}\int_{R}{\left[\int_{R^{d}}\partial_{x_{i}}\phi \left(x,v\right)u\left(\cdot ,x,v\right)dx,M_{i}\left(\cdot ,v\right)\right]}_{t}dv\\ = & 2\sum_{i=1}^{d}\sum_{j=1}^{d}\int_{0}^{t}ds\int_{R^{d+1}}a_{i,j}\left(s,v\right)\partial_{x_{i},x_{j}}^{2}\phi \left(x,v\right)u\left(s,x,v\right)dxdv. \end{array}$$

Noticing that whichsoever (11) or (25) holds, then for every $\phi \in D(R^{d+1})$, and for all t∈[0,∞), the martingale part of $\int_{R^{d}}\partial_{x_{i}}\phi \left(x,v\right)u\left(t,x,v\right)dx$ (1⩽i⩽d) is given by

$$\sum_{j=1}^{d}\int_{0}^{t}\int_{R^{d}}\partial_{x_{i},x_{j}}^{2}\phi \left(x,v\right)u\left(s,x,v\right)dxM_{j}\left(ds,v\right).$$

Therefore

$$\begin{array}{cc} & \sum_{i=1}^{d}\int_{R}{\left[\int_{R^{d}}\partial_{x_{i}}\phi \left(x,v\right)u\left(\cdot ,x,v\right)dx,M_{i}\left(\cdot ,v\right)\right]}_{t}dv\\ = & \sum_{i=1}^{d}\sum_{j=1}^{d}\int_{R}{\left[\int_{0}^{\cdot }M_{j}\left(ds,v\right)\int_{R^{d}}\partial_{x_{i},x_{j}}^{2}\phi \left(x,v\right)u\left(s,x,v\right)dx,M_{i}\left(\cdot ,v\right)\right]}_{t}dv\\ = & \sum_{i=1}^{d}\sum_{j=1}^{d}\sum_{k=1}^{n}\int_{R}dv\int_{0}^{t}ds\int_{R^{d}}\partial_{x_{i},x_{j}}^{2}\phi \left(x,v\right)u\left(s,x,v\right)\sigma_{i,k}\left(s,v\right)\sigma_{j,k}\left(s,v\right)dx\\ = & 2\sum_{i=1}^{d}\sum_{j=1}^{d}\int_{0}^{t}ds\int_{R^{d+1}}a_{i,j}\left(s,v\right)\partial_{x_{i},x_{j}}^{2}\phi \left(x,v\right)u\left(s,x,v\right)dxdv. \end{array}$$

The proof of Lemma 1 is complete. □

**Lemma** **2.** *For every* p∈[1,∞] *, we have the following embedding:*

$$L^{p}\left(R^{d};W_{loc}^{1,1}\left(R\right)\right)↪L^{p}\left(R^{d};C\left(R\right)\right).$$

**Proof.**  Clearly, $W_{loc}^{1,1}\left(R\right)↪C\left(R\right)$ (see [33]), for any $g\in L^{p}\left(R^{d};W_{loc}^{1,1}\left(R\right)\right)$, g(x,·)∈C(R) for almost everywhere $x\in R^{d}$. Let −∞<a<b<∞ be two real numbers.

When p<∞,

$$\begin{array}{ccc} {∥g∥}_{L^{p}\left(R^{d};L^{\infty }\left(a,b\right)\right)}^{p} & = & \int_{R^{d}}{∥g\left(x,\cdot \right)∥}_{L^{\infty }\left(a,b\right)}^{p}dx\\ & = & \int_{R^{d}}{∥\int_{a}^{\cdot }\partial_{v}g\left(x,v\right)dv+g\left(x,a\right)∥}_{L^{\infty }\left(a,b\right)}^{p}dx\\ & ⩽ & 2^{p-1}\{\int_{R^{d}}{\left[\int_{a}^{b}\left|\partial_{v}g\left(x,v\right)\right|dv\right]}^{p}dx+\int_{R^{d}}{\left|g\left(x,a\right)\right|}^{p}dx\}<\infty . \end{array}$$

When p=∞, for almost everywhere $x\in R^{d}$, and all v∈[a,b],

$$\left|f\left(x,v\right)\right|=|\int_{a}^{v}\partial_{y}g\left(x,y\right)dy+g\left(x,a\right)|⩽\int_{a}^{b}\left|\partial_{y}g\left(x,y\right)\right|dy+\left|g\left(x,a\right)\right|<\infty ,$$

which hints

$$L^{p}\left(R^{d};W_{loc}^{1,1}\left(R\right)\right)↪L^{p}\left(R^{d};L_{loc}^{\infty }\left(R\right)\right).$$

Thus the desired result follows. □

In order to prove the uniqueness of the stochastic entropy solution, we need another two lemmas below, the first one follows from DiPerna and Lions [34], and the proof is analogue, we only give the details for the second one.

**Lemma** **3.** *Let* k∈N *,* T∈(0,∞) *,* $1⩽p_{1},p_{2},q_{1},q_{2},\alpha ,\beta ⩽\infty$ *, that* $E\in L^{p_{1}}\left(\Omega ;L^{p_{2}}\left(0,T;W_{loc}^{1,\alpha }\left(R^{k};R^{k}\right)\right)\right)$ *,* $G\in L^{q_{1}}\left(\Omega ;L^{q_{2}}\left(0,T;L_{loc}^{\beta }\left(R^{k}\right)\right)\right)$ *. Then*

$$\left(E\cdot \nabla G\right)*{\tilde{\varrho }}_{\varepsilon_{1}}-E\cdot \nabla \left(G*{\tilde{\varrho }}_{\varepsilon_{1}}\right)⟶0\;in\;L^{r_{1}}\left(\Omega ;L^{r_{2}}\left(0,T;L_{loc}^{\gamma }\left(R^{k}\right)\right)\right)\;as\;\varepsilon_{1}\to 0,$$

*where* $1⩽\gamma ,r_{1},r_{2}<\infty$ *, satisfying*

$$\frac{1}{\alpha }+\frac{1}{\beta }⩽\frac{1}{\gamma },\;\frac{1}{p_{1}}+\frac{1}{q_{1}}⩽\frac{1}{r_{1}},\;\frac{1}{p_{2}}+\frac{1}{q_{2}}⩽\frac{1}{r_{2}},$$

*and*

$${\tilde{\varrho }}_{\varepsilon_{1}}=\frac{1}{{\varepsilon_{1}}^{k}}\tilde{\varrho }\left(\frac{\cdot }{\varepsilon_{1}}\right)\;with\;\tilde{\varrho }\in D_{+}\left(R^{k}\right),\;\int_{R^{k}}\tilde{\varrho }\left(y\right)dy=1,\;\varepsilon_{1}>0.$$

*And when* k=d *, we set* $\tilde{\varrho }$ *by* $\varrho_{1}$ *.*

**Lemma** **4.** *Let* $g\in L^{2}(\Omega ;L_{loc}^{2}\left(\left[0,\infty \right)\right)$ *, then*

$$[\int_{0}^{\cdot }g\left(s\right)dW\left(s\right)*\varrho_{2,\varepsilon_{2}}]\left(t\right)⟶\int_{0}^{t}g\left(s\right)dW\left(s\right),\;in\;L^{2}(\Omega ;L_{loc}^{2}\left(\left[0,\infty \right)\right)\;as\;\varepsilon_{2}\to 0,$$

*where* W(t) *is a 1-dimensional standard Wiener process, and*

$$\varrho_{2,\varepsilon_{2}}=\frac{1}{\varepsilon_{2}}\varrho_{2}\left(\frac{\cdot }{\varepsilon_{2}}\right)\;\varrho_{2}\in D_{+}\left(R\right),\;\int_{R}\varrho_{2}\left(t\right)dt=1,\;supp\varrho_{2}\subset \left(-1,0\right).$$

**Proof.**  In fact, for every T∈(0,∞),

E∫0T|[∫0·g(s)dW(s)∗ϱ2,ε2](t)−∫0tg(s)dW(s)|2dt=E∫0T|∫Rϱ2,ε2(s)ds∫0t−sg(r)dW(r)−∫0tg(r)dW(r)|2dt=E∫0T|∫−10ϱ2(s)ds∫0t−ε2sg(r)dW(r)−∫0tg(r)dW(r)|2dt=E∫0T|∫−10ϱ2(s)ds∫tt−ε2sg(r)dW(r)|2dt(26)⩽∫0TEsups∈[0,1]|∫tt+ε2sg(r)dW(r)|2dt.

For $g\in L^{2}(\Omega ;L_{loc}^{2}\left(\left[0,\infty \right)\right)$, the stochastic process $\left\{\int_{0}^{t}g\left(r\right)dW_{r},\;t⩾0\right\}$ is a martingale. With the help of Doob’s inequality and the Itô isometry (see [35]), from (26), one obtains

E∫0T|[∫0·g(s)dW(s)∗ϱ2,ε2](t)−∫0tg(s)dW(s)|2dt(27)⩽4∫0Tsup0⩽s⩽1E|∫tt+ε2sg(r)dW(r)|2dt=4∫0T∫tt+ε2E|g(r)|2drdt.

By letting $\varepsilon_{2}$ tend to 0 in (27), we finish the proof. □

## 3. Proof of Theorem 1

For every ζ,ϑ∈R,

$$\int_{R}|\chi_{\zeta }\left(v\right)-\chi_{\vartheta }\left(v\right)|dv=|\zeta -\vartheta |,$$

so (10) implies (15), and vice versa. We need to check the rest of (i) and (ii) in Theorem 1.

Let ρ be a stochastic entropy solution of (1), (2) fulfilling the statement (i) in Theorem 1. For every v∈R, it renders that

(28) $$\partial_{t}\eta \left(\rho ,v\right)+{\mathrm{div}}_{x}Q\left(\rho ,v\right)+\sum_{i=1}^{d}\sum_{j=1}^{n}\partial_{x_{i}}Q_{i,j}\left(t,\rho ,v\right)\circ {\dot{W}}_{j}\left(t\right)=\mathrm{sgn}\left(\rho -v\right)A\left(t,x,\rho \right)-2m,$$

for almost all ω∈Ω, where

(29) $$\left\{ \begin{array}{c} \eta (\rho ,v)=|\rho -v|-|v|,\\ Q(\rho ,v)=\mathrm{sgn}(\rho -v)[F(\rho )-F(v)]-\mathrm{sgn}(v)F(v),\\ Q_{i,j}\left(t,\rho ,v\right)=\mathrm{sgn}\left(\rho -v\right)\left[B_{i,j}\left(t,\rho \right)-B_{i,j}\left(t,v\right)\right]\\ \;-\mathrm{sgn}\left(v\right)B_{i,j}\left(t,v\right),\;1⩽i⩽d,1⩽j⩽n,\\ m\;\mathrm{is}\;a\;\mathrm{nonnegative}\;\mathrm{measure}\;\mathrm{on}\;\left[0,\infty \right)\times R^{d+1}. \end{array}\right.$$

For every $\phi_{1}\in D\left(R\right)$, then

$${\langle \partial_{v}\mathrm{sgn}\left(\rho -v\right)A\left(t,x,\rho \right),\;\phi_{1}\rangle }_{v}=-2\phi_{1}\left(\rho \right)A\left(t,x,\rho \right).$$

Observing that

(30) $$\int_{R}g^{\prime }\left(v\right)u\left(t,x,v\right)dv=g\left(\rho \left(t,x\right)\right)-g\left(0\right),\;\mathrm{for}\;\mathrm{every}\;g\in W_{loc}^{1,1}\left(R\right),$$

and A(t,x,0)=0. On account of (4), it follows that

$${\langle \partial_{v}u\left(t,x,\cdot \right)A\left(t,x,\cdot \right),\;\phi_{1}\rangle }_{v}=-\int_{R}u\left(t,x,v\right)\partial_{v}\left(\phi_{1}\left(v\right)A\left(t,x,v\right)\right)dv=-\phi_{1}\left(\rho \right)A\left(t,x,\rho \right),$$

thus $\partial_{v}\mathrm{sgn}\left(\rho -v\right)A\left(t,x,\rho \right)=2\partial_{v}u\left(t,x,v\right)A\left(t,x,v\right)$.

Similarly, by using conditions (3) and (5), one computes in the sense of distributions that

(31) $$\left\{ \begin{array}{c} \partial_{v}\eta \left(\rho ,v\right)=-2u\left(t,x,v\right),\;\partial_{v}Q\left(\rho ,v\right)=-2f\left(v\right)u\left(t,x,v\right),\\ \partial_{v}Q_{i,j}\left(t,\rho ,v\right)=-2\sigma_{i,j}\left(t,v\right)u\left(t,x,v\right),\;1⩽i⩽d,1⩽j⩽n. \end{array}\right.$$

From (31), one derives the identity (11). In order to prove the assertion of Theorem 1 (i), it suffices to show that *m* satisfies all the properties described in (i).

Noting that ρ is bounded local-in-time, from (28) and (29), for every fixed T>0, and almost all ω∈Ω, *m* is supported in $\left[0,T\right]\times R^{d}\times \left[-K,K\right]$, with $K={∥\rho ∥}_{L^{\infty }\left(\left(0,T\right)\times R^{d}\times \Omega \right)}$. Accordingly, it remains to examine that *m* is bounded and continuous in *t*. And it is sufficient to show that $m(\left[0,t\right]\times R^{d+1})$ is bounded and continuous in *t*.

Since m⩾0 and it is supported in a compact subset for *v* in R, we obtain

$$0⩽{\langle m,\;\psi ⊗1\rangle }_{t,x,v}=-{\langle \partial_{t}u+f\left(v\right)\cdot \nabla_{x}u+A\partial_{v}u+\sum_{i=1}^{d}\partial_{x_{i}}u\circ {\dot{M}}_{i}\left(t,v\right),\;\psi ⊗v\rangle }_{t,x,v},\;P-a.s.,$$

for every $\psi \in D_{+}\left(\left[0,\infty \right)\times R^{d}\right)$.

By Lemma 1, then

0⩽〈m, ψ⊗1〉t,x,v=−〈∂tu+f(v)·∇xu+A∂vu+∑i=1d∑j=1nσi,j(t,v)∂xiuW˙j(t), ψ⊗v〉t,x,v(32)+∑i=1d∑j=1d〈ai,j(t,v)∂xi,xj2u, ψ⊗v〉t,x,v.

Thanks to (30),

$$\begin{array}{cc} & -{\langle \partial_{t}u+f\left(v\right)\cdot \nabla_{x}u+A\partial_{v}u+\sum_{i=1}^{d}\sum_{j=1}^{n}\sigma_{i,j}\left(t,v\right)\partial_{x_{i}}u{\dot{W}}_{j}\left(t\right),\;\psi ⊗v\rangle }_{t,x,v}\\ & +\sum_{i=1}^{d}\sum_{j=1}^{d}{\langle a_{i,j}\left(t,v\right)\partial_{x_{i},x_{j}}^{2}u,\;\psi ⊗v\rangle }_{t,x,v}\\ = & \frac{1}{2}\int_{0}^{T}\int_{R^{d}}\partial_{t}\psi \left(t,x\right)\rho^{2}dxdt+\frac{1}{2}\int_{R^{d}}\psi \left(0,x\right)\rho_{0}^{2}\left(x\right)dx+\int_{0}^{T}\int_{R^{d}}\rho A\left(t,x,\rho \right)\psi \left(t,x\right)dxdt\\ & +\int_{0}^{T}\int_{R^{d}}[\rho \left(t,x\right)F\left(\rho \left(t,x\right)\right)-\int_{0}^{\rho (t,x)}F\left(v\right)dv]\cdot \nabla_{x}\psi \left(t,x\right)dxdt\\ & +\sum_{i=1}^{d}\sum_{j=1}^{d}\int_{0}^{T}\int_{R^{d}}[A_{i,j}\left(t,\rho \left(t,x\right)\right)\rho \left(t,x\right)-\int_{0}^{\rho (t,x)}A_{i,j}\left(t,v\right)dv]\partial_{x_{i},x_{j}}^{2}\psi \left(t,x\right)dxdt\\ & +\sum_{i=1}^{d}\sum_{j=1}^{n}\int_{0}^{T}\int_{R^{d}}[B_{i,j}\left(t,\rho \right)\rho \left(t,x\right)-\int_{0}^{\rho (t,x)}B_{i,j}\left(t,v\right)dv]\partial_{x_{i}}\psi \left(t,x\right)dxdW_{j}\left(t\right), \end{array}$$

for every T>0 and $\psi \in D_{+}\left(\left[0,T\right)\times R^{d}\right)$, where $A_{i,j}\left(t,v\right)=\int_{0}^{v}a_{i,j}\left(t,r\right)dr$.

On account of Hypotheses (3)–(5), by using Lemma 2, it leads to

〈m, ψ⊗1〉t,x,v⩽12∫0T∫Rd∂tψρ2dxdt+12∫Rdψ(0,x)ρ02dx+C(T)∫0T∫Rda˜(t,x)|ρ(t,x)|ψ(t,x)dxdt+C(T)∫0T∫Rd|ρ(t,x)||∇xψ(t,x)|dxdt+C(T)∑i=1d∑j=1d∫0T∫Rda˜i,j(t)|ρ(t,x)||∂xi,xj2ψ(t,x)|dxdt(33)+∑i=1d∑j=1n∫0T∫Rd[Bi,j(t,ρ)ρ−∫0ρ(t,x)Bi,j(t,v)dv]∂xiψ(t,x)dxdWj(t),

where

$$\begin{array}{c} \tilde{a}\left(t,x\right)=\underset{v\in [-K,K]}{\sup}\left|A\left(t,x,v\right)\right|\in L_{loc}^{1}\left(\left[0,\infty \right);L^{1}\left(R^{d}\right)\right)+L_{loc}^{1}\left(\left[0,\infty \right);L^{\infty }\left(R^{d}\right)\right),\\ {\tilde{a}}_{i,j}\left(t\right)\;=\underset{v\in [-K,K]}{\sup}\left|A_{i,j}\left(t,v\right)\right|\in L_{loc}^{1}\left(\left[0,\infty \right)\right). \end{array}$$

Using the Itô isometry and Lemma 1,

E{∑i=1d∑j=1n∫0T∫Rd[Bi,j(t,ρ)ρ−∫0ρ(t,x)Bi,j(t,v)dv]∂xiψ(t,x)dxdWj(t)}2=∫0T∑j=1nE[∑i=1d∫Rd[Bi,j(t,ρ)ρ−∫0ρ(t,x)Bi,j(t,v)dv]∂xiψ(t,x)dx]2dt(34)⩽C∑i=1d∑j=1nE∫0Tb˜i,j2(t)[∫Rd|ρ(t,x)||∂xiψ(t,x)|dx]2dt,

where ${\tilde{b}}_{i,j}\left(t\right)\;=\sup_{v\in [-K,K]}\left|B_{i,j}\left(t,v\right)\right|\in L_{loc}^{2}\left(\left[0,\infty \right)\right).$

Obviously, (33) holds ad hoc for $\psi \left(t,x\right)=\psi_{1}\left(t\right)\theta_{k_{1}}\left(x\right)$, where $k_{1}\in N$, $\psi_{1}\in D_{+}\left(\left[0,T\right)\right),\theta \in D_{+}\left(R^{d}\right)$,

(35) $$\theta_{k_{1}}\left(x\right)=\theta \left(\frac{x}{k_{1}}\right),\;\theta \left(x\right)=\{\begin{array}{c} 1,\;\mathrm{when}\;|x|⩽1,\\ 0,\;\mathrm{when}\;|x|>2. \end{array}$$

For this fixed $k_{1}$, by an approximation demonstration, one can fetch

$$\psi_{1}\left(t\right)=\{\begin{array}{cc} 1, & t\in [0,T-\frac{1}{k_{1}}],\\ -k_{1}\left(t-T\right), & t\in \left(T-\frac{1}{k_{1}},T\right],\\ 0, & t\in (T,\infty ). \end{array}$$

By letting $k_{1}\to \infty$, we gain from (33) and (34) (by choosing a subsequence if necessary), that

∫0T∫Rd+1m(dt,dx,dv)(36)⩽12[∫Rdρ02dx−∫Rdρ2(T,x)dx]+C(T)∫0T∫Rda˜(t,x)|ρ(t,x)|dxdt, P−a.s.,

which suggests that for every given T>0, *m* is bounded on $\left[0,T\right]\times R^{d+1}$ and $m\in L^{1}\left(\Omega ;D^{\prime }\left(\left[0,\infty \right)\times R^{d+1}\right)\right)$.

Specially, when T→0, we obtain

$$\lim_{T\to 0}\int_{0}^{T}\int_{R^{d+1}}m\left(dt,dx,dv\right)=0,\;P-a.s..$$

The arguments employed above for 0 and *T* adapted to every 0⩽s,t<∞ now, yields that

$$\lim_{t\to s}\int_{s}^{t}\int_{R^{d+1}}m\left(dr,dx,dv\right)=0,$$

which hints *m* is continuous in *t*. So *u* is a stochastic weak solution of (11)–(13) with *m* satisfying (14).

Let us show the reverse fact. Since *m* satisfies (14) and $u\left(t,x,v\right)=\chi_{\rho (t,x)}\left(v\right)$ solves (11)–(13), for every $\psi \in D(R^{d})$, then $\int_{R^{d}}\psi \left(x\right)\rho \left(t,x\right)dx=\int_{R^{d+1}}\psi \left(x\right)u\left(t,x,v\right)dxdv$ is $F_{t}$-adapted. It remains to show the inequality (9).

Given ϵ>0 and $\bar{\rho }\in R$, set

$$\eta_{\epsilon }\left(r,\bar{\rho }\right)=\left(\sqrt{{\left(r-\bar{\rho }\right)}^{2}+\epsilon^{2}}-\epsilon \right)-\left|\bar{\rho }\right|\in C^{2}\left(R\right),$$

then $\eta_{\epsilon }$ is convex, $\eta_{\epsilon }^{\prime }\left(r,\bar{\rho }\right)\in C_{b}\left(R\right)$, and

$$\eta_{\epsilon }\left(r,\bar{\rho }\right)⟶|r-\bar{\rho }|-|\bar{\rho }|\;\mathrm{as}\;\epsilon ⟶0.$$

In a consequence of u(t,x,v) solving (11)–(13) with *m* satisfying (14), it follows that

〈∂vm, ψηϵ′(v,ρ¯)ξk2(v)〉t,x,v=〈∂tu+∑i=1d∂xiu∘M˙i(t,v), ψηϵ′(v,ρ¯)ξk2(v)〉t,x,v+〈f(v)·∇xu+A∂vu, ψηϵ′(v,ρ¯)ξk2(v)〉t,x,v=〈∂tu+∑i=1d∑j=1nσi,j(t,v)∂xiu∘W˙j(t), ψηϵ′(v,ρ¯)ξk2(v)〉t,x,v(37)+〈f(v)·∇xu+A∂vu, ψηϵ′(v,ρ¯)ξk2(v)〉t,x,v,

for every $\psi \in D_{+}\left(\left[0,\infty \right)\times R^{d}\right)$, $\xi \in D_{+}\left(R\right)$, $k_{2}\in N$, where

(38) $$\xi_{k_{2}}\left(v\right)=\xi \left(\frac{v}{k_{2}}\right),\;0⩽\xi ⩽1,\;\xi \left(v\right)=\{\begin{array}{cc} 1, & \mathrm{when}\;|v|⩽1,\\ 0, & \mathrm{when}\;|v|⩾2. \end{array}$$

Applying the partial integration, one deduces

(39) $$\lim_{k\to \infty }{\langle \partial_{v}m,\;\psi \eta_{\epsilon }^{\prime }\left(v,\bar{\rho }\right)\xi_{k_{2}}\rangle }_{t,x,v}=-\lim_{k\to \infty }{\langle m,\;\psi \left[\eta_{\epsilon }^{\prime \prime }\left(v,\bar{\rho }\right)\xi_{k_{2}}+\eta_{\epsilon }^{\prime }\left(v,\bar{\rho }\right)\xi_{k_{2}}^{\prime }\right]\rangle }_{t,x,v}⩽0,\;P-a.s.,$$

when $k_{2}$ is large enough, for *m* yields the properties stated in Theorem 1 (i).

Upon using (30) and (39), from (37), we derive

∫0∞dt∫Rd∂tψ(t,x)[ηϵ(ρ,ρ¯)−ηϵ(0,ρ¯)]dx+∫0∞dt∫RdQϵ(ρ,ρ¯)·∇xψdx⩾−∫Rdψ(0,x)[ηϵ(ρ0,ρ¯)−ηϵ(0,ρ¯)]dx−∫0∞dt∫Rdηϵ′(ρ,ρ¯)A(t,x,ρ)ψ(t,x)dx(40)−∑i=1d∑j=1n∫0∞∘dWj∫Rd∂xiψQi,jϵ(t,ρ,ρ¯)dx,

by taking $k_{2}$ to infinity, here

$$Q_{\epsilon }\left(\rho ,\bar{\rho }\right)=\int_{R}f\left(v\right)\eta_{\epsilon }^{\prime }\left(v,\bar{\rho }\right)u\left(t,x,v\right)dv,\;Q_{i,j}^{\epsilon }\left(t,\rho ,\bar{\rho }\right)=\int_{R}\sigma_{i,j}\left(t,v\right)\eta_{\epsilon }^{\prime }\left(v,\bar{\rho }\right)u\left(t,x,v\right)dv.$$

On the other hand

$$\lim_{\epsilon \to 0}\eta_{\epsilon }^{\prime }\left(\rho ,\bar{\rho }\right)=\mathrm{sgn}\left(\rho -\bar{\rho }\right)$$

and

$$\lim_{\epsilon \to 0}Q_{\epsilon }\left(\rho ,\bar{\rho }\right)=\mathrm{sgn}\left(\rho -\bar{\rho }\right)\left[F\left(\rho \right)-F\left(\bar{\rho }\right)\right]-\mathrm{sgn}\left(\bar{\rho }\right)\left[F\left(\bar{\rho }\right)-F\left(0\right)\right],$$

$$\lim_{\epsilon \to 0}Q_{i,j}^{\epsilon }\left(t,\rho ,\bar{\rho }\right)=\mathrm{sgn}\left(\rho -\bar{\rho }\right)\left[B_{i,j}\left(t,\rho \right)-B_{i,j}\left(t,\bar{\rho }\right)\right]-\mathrm{sgn}\left(\bar{\rho }\right)\left[B_{i,j}\left(t,\bar{\rho }\right)-B_{i,j}\left(t,0\right)\right],$$

for almost everywhere $\left(\omega ,t,x\right)\in \Omega \times \left[0,\infty \right)\times R^{d}$.

If one lets ϵ approach to zero in (40), we attain the inequality (9), thus ρ is a stochastic entropy solution.

**Remark** **4.** *Our proof for Theorem 1 is inspired by Theorem 1 in [36], but the demonstration here appears to be finer, and for more details, one can see [36] and also see [37] for nonlocal conservation laws.*

## 4. Proofs of Theorem 2 and Corollary 1

We begin our discussion in this section to prove Theorem 2. Let $\rho_{1}$ and $\rho_{2}$ be two stochastic entropy solutions of (1), with initial values $\rho_{0,1}$ and $\rho_{0,2}$, respectively. Then $u_{1}=\chi_{\rho_{1}}$ and $u_{2}=\chi_{\rho_{2}}$ are stochastic weak solutions of (11) with nonhomogeneous terms $\partial_{v}m_{1}$ and $\partial_{v}m_{2}$, initial datum $u_{0,1}=\chi_{\rho_{0,1}}$ and $u_{0,2}=\chi_{\rho_{0,2}}$, respectively.

Let $\varrho_{1}$ and $\varrho_{2}$ be two regularization kernels described in Lemmas 3 and 4, respectively. Let $\varrho_{3}$ be another regularization kernel in variable *v*, i.e.,

$$\varrho_{3}\in D_{+}\left(R\right),\;\int_{R}\varrho_{3}\left(v\right)dv=1.$$

For $\varepsilon_{1},\varepsilon_{2},\epsilon >0$, set

$$\varrho_{1,\varepsilon_{1}}\left(x\right)=\frac{1}{\varepsilon_{1}^{d}}\varrho_{1}\left(\frac{x}{\varepsilon_{1}}\right),\;\varrho_{2,\varepsilon_{2}}\left(t\right)=\frac{1}{\varepsilon_{2}}\varrho_{2}\left(\frac{t}{\varepsilon_{2}}\right),\;\varrho_{3,\epsilon }\left(v\right)=\frac{1}{\epsilon }\varrho_{3}\left(\frac{v}{\epsilon }\right),$$

then $u_{\iota }^{\varepsilon ,\epsilon }:=u_{\iota }*\varrho_{1,\varepsilon_{1}}*\varrho_{2,\varepsilon_{2}}*\varrho_{3,\epsilon }$ (ι=1,2) yields that

(41) $$\left\{ \begin{array}{c} \partial_{t}u_{\iota }^{\varepsilon ,\epsilon }+f\left(v\right)\cdot \nabla_{x}u_{\iota }^{\varepsilon ,\epsilon }+A\left(t,x,v\right)\partial_{v}u_{\iota }^{\varepsilon ,\epsilon }\\ \;+\sum_{i=1}^{d}\partial_{x_{i}}u_{\iota }^{\varepsilon ,\epsilon }\circ {\dot{M}}_{i}\left(t,v\right)=\partial_{v}m_{\iota }^{\varepsilon ,\epsilon }+R_{\iota }^{\varepsilon ,\epsilon },\\ u_{\iota }^{\varepsilon ,\epsilon }{\left(t,x,v\right)|}_{t=0}=\chi_{\rho_{0}^{\iota }}*\varrho_{1,\varepsilon_{1}}*\varrho_{3,\epsilon }\left(x,v\right), \end{array}\right.$$

here $R_{\iota }^{\varepsilon ,\epsilon }=R_{\iota ,1}^{\varepsilon ,\epsilon }+R_{\iota ,2}^{\varepsilon ,\epsilon }+R_{\iota ,3}^{\varepsilon ,\epsilon }$, and

(42) $$\left\{ \begin{array}{c} R_{\iota ,1}^{\varepsilon ,\epsilon }=f\left(v\right)\cdot \nabla_{x}u_{\iota }^{\varepsilon ,\epsilon }-{\left[f\left(v\right)\cdot \nabla_{x}u_{\iota }\right]}^{\varepsilon ,\epsilon },\\ R_{\iota ,2}^{\varepsilon ,\epsilon }=A\left(t,x,v\right)\partial_{v}u_{\iota }^{\varepsilon ,\epsilon }-{\left[A\left(t,x,v\right)\partial_{v}u_{\iota }\right]}^{\varepsilon ,\epsilon },\\ R_{\iota ,3}^{\varepsilon ,\epsilon }=\sum_{i=1}^{d}\{\partial_{x_{i}}u_{\iota }^{\varepsilon ,\epsilon }\circ {\dot{M}}_{i}\left(t,v\right)-{\left[\partial_{x_{i}}u_{\iota }\circ {\dot{M}}_{i}\left(t,v\right)\right]}^{\varepsilon ,\epsilon }\}. \end{array}\right.$$

For every δ>0, we set $\eta_{\delta }\left(u\right)={\left(u^{2}+\delta \right)}^{\frac{1}{2}}$. For ι=1,2, if one uses Itô’s formula for $\eta_{\delta }\left(u_{\iota }^{\varepsilon ,\epsilon }\right)$ first, and lets δ tend to 0 next, it follows that

ddt∫Rd+1|uιε,ϵ(t,x,v)|ξk2(v)θk1(x)dxdv=∫Rd+1|uιε,ϵ|ξk2(v)f(v)·∇xθk1(x)dxdv+∫Rd+1|uιε,ϵ(t,x,v)|∂v[ξk2(v)A(t,x,v)]θk1(x)dxdv+∑i=1d∫Rd+1|uιε,ϵ|∂xiθk1(x)ξk2(v)∘M˙i(t,v)dxdv+∫Rd+1sgn(uιε,ϵ)ξk2(v)θk1(x)Rιε,ϵdxdv(43)+∫Rd+1ξk2(v)sgn(uιε,ϵ)θk1(x)∂vmιε,ϵ(t,x,v)dxdv,

where $M_{i}$, $\theta_{k_{1}}$ and $\xi_{k_{2}}$ are given by (13), (35) and (38), respectively.

Analogue calculations also yield that

ddt∫Rd+1u1ε,ϵ(t,x,v)u2ε,ϵ(t,x,v)ξk2(v)θk1(x)dxdv=∫Rd+1u1ε,ϵ(t,x,v)u2ε,ϵ(t,x,v)ξk2(v)f(v)·∇xθk1(x)dxdv+∫Rd+1u1ε,ϵ(t,x,v)u2ε,ϵ(t,x,v)∂v[ξk2(v)A(t,x,v)]θk1(x)dxdv+∑i=1d∫Rd+1u1ε,ϵ(t,x,v)u2ε,ϵ(t,x,v)∂xiθk1ξk2∘M˙i(t,v)dxdv+∫Rd+1ξk2θk1[u1ε,ϵ(t,x,v)∂vm2ε,ϵ+u2ε,ϵ(t,x,v)∂vm1ε,ϵ]dxdv(44)+∫Rd+1ξk2(v)θk1(x)[R1ε,ϵ(t,x,v)u2ε,ϵ(t,x,v)+R2ε,ϵ(t,x,v)u1ε,ϵ(t,x,v)]dxdv.

From (43) and (44), one infers

ddt∫Rd+1[|u1ε,ϵ(t,x,v)|+|u2ε,ϵ(t,x,v)|−2u1ε,ϵ(t,x,v)u2ε,ϵ(t,x,v)]ξk2(v)θk1(x)dxdv=∫Rd+1[|u1ε,ϵ(t,x,v)|+|u2ε,ϵ(t,x,v)|−2u1ε,ϵ(t,x,v)u2ε,ϵ(t,x,v)]ξk2(v)f(v)·∇xθk1(x)dxdv+∫Rd+1[|u1ε,ϵ(t,x,v)|+|u2ε,ϵ(t,x,v)|−2u1ε,ϵ(t,x,v)u2ε,ϵ(t,x,v)]∂v[ξk2A(t,x,v)]θk1dxdv+∑i=1d∫Rd+1[|u1ε,ϵ(t,x,v)|+|u2ε,ϵ(t,x,v)|−2u1ε,ϵ(t,x,v)u2ε,ϵ(t,x,v)]∂xiθk1(x)× ξk2(v)∘M˙i(t,v)dxdv+∫Rd+1ξk2(v)θk1(x)[sgn(u1ε,ϵ)R1ε,ϵ+sgn(u2ε,ϵ)R2ε,ϵ]dxdv(45)−2∫Rd+1ξk2(v)θk1(x)[R1ε,ϵu2ε,ϵ+R2ε,ϵu1ε,ϵ]dxdv+I(t),

where

$$\begin{array}{ccc} I(t) & = & \int_{R^{d+1}}\xi_{k_{2}}\left(v\right)\theta_{k_{1}}\left(x\right)\left[\mathrm{sgn}\left(u_{1}^{\varepsilon ,\epsilon }\right)\partial_{v}m_{1}^{\varepsilon ,\epsilon }+\mathrm{sgn}\left(u_{2}^{\varepsilon ,\epsilon }\right)\partial_{v}m_{2}^{\varepsilon ,\epsilon }\right]dxdv\\ & & -2\int_{R^{d+1}}\xi_{k_{2}}\left(v\right)\theta_{k_{1}}\left(x\right)\left[u_{1}^{\varepsilon ,\epsilon }\partial_{v}m_{2}^{\varepsilon ,\epsilon }+u_{2}^{\varepsilon ,\epsilon }\partial_{v}m_{1}^{\varepsilon ,\epsilon }\right]dxdv=:I_{1}\left(t\right)-2I_{2}\left(t\right). \end{array}$$

Observing that for every T>0, and almost all ω∈Ω, $m_{1}$ and $m_{2}$ are bounded on $\left[0,T\right]\times R^{d+1}$, supported in $\left[0,T\right]\times R^{d}\times \left[-K,K\right]$, where

(46) $$K=\max\{{∥\rho_{1}∥}_{L^{\infty }\left(\Omega \times \left(0,T\right)\times R^{d}\right)},\;{∥\rho_{2}∥}_{L^{\infty }\left(\Omega \times \left(0,T\right)\times R^{d}\right)}\}.$$

Thus by taking $k_{2}>K$,

(47) $$\left\{ \begin{array}{c} \int_{R^{d+1}}\partial_{v}\left(\xi_{k_{2}}\left(v\right)\right)\theta_{k_{1}}\left(x\right)\left[\mathrm{sgn}\left(u_{1}^{\varepsilon ,\epsilon }\right)m_{1}^{\varepsilon ,\epsilon }+\mathrm{sgn}\left(u_{2}^{\varepsilon ,\epsilon }\right)m_{2}^{\varepsilon ,\epsilon }\right]dxdv=0,\\ \int_{R^{d+1}}\partial_{v}\left(\xi_{k_{2}}\left(v\right)\right)\theta_{k_{1}}\left(x\right)\left[u_{1}^{\varepsilon ,\epsilon }m_{2}^{\varepsilon ,\epsilon }+u_{2}^{\varepsilon ,\epsilon }m_{1}^{\varepsilon ,\epsilon }\right]dxdv=0. \end{array}\right.$$

From (41), with the aid of assumptions (3)–(5) and Lemma 2, $m_{\iota }^{\varepsilon }\;\left(\iota =1,2\right)$ is continuous in *v* in a neighborhood of zero. Besides, for almost everywhere (t,x,v),

$$\mathrm{sgn}\left(u_{\iota }^{\varepsilon ,\epsilon }\right)⟶\mathrm{sgn}\left(u_{\iota }^{\varepsilon }\right)=\mathrm{sgn}\left(v\right),\;\mathrm{as}\;\epsilon \to 0.$$

Hence for large $k_{2}$ ($k_{2}>K$) and every t⩾0,

(48) $$\lim_{\epsilon \to 0}I_{1}\left(t\right)=-2\int_{R^{d}}\theta_{k_{1}}\left(x\right)\left[m_{1}^{\varepsilon }\left(t,x,0\right)+m_{2}^{\varepsilon }\left(t,x,0\right)\right]dx.$$

Moreover, due to (30) and the fact $m_{\iota }⩾0\;\left(\iota =1,2\right)$, if one chooses $k_{2}$ large enough, then

I2(t)=∫Rd+1{∫Rd+2[ξk2(ρ1(t−s,x−y)+τ)m2ε,ϵ(t,x,ρ1(t−s,x−y)+τ)    +ξk2(ρ2(t−s,x−y)+τ)m1ε,ϵ(t,x,ρ2(t−s,x−y)+τ)]      × ϱ3,ϵ(τ)θk1(x)dxdvdτ}ϱ1,ε1(y)ϱ2,ε2(s)dyds−∫Rd+1ξk2(v)θk1(x)[m1ε,ϵ(t,x,v)+m2ε,ϵ(t,x,v)]ϱ3,ϵ(v)dxdv⩾−∫Rd+1ξk2(v)θk1(x)[m1ε,ϵ(t,x,v)+m2ε,ϵ(t,x,v)]ϱ3,ϵ(v)dxdv(49)⟶−∫Rdθk1(x)[m1ε(t,x,0)+m2ε(t,x,0)]dx, as ϵ→0.

On account of (42), thanks to conditions (3) and (16), and Lemma 3, then,

(50) $$\lim_{\varepsilon_{2}\to 0}\lim_{\epsilon \to 0}R_{\iota ,1}^{\varepsilon ,\epsilon }=0,\;\mathrm{in}\;L^{1}\left(\Omega ;L^{1}\left(0,T;L_{loc}^{1}\left(R^{d+1}\right)\right)\right),\;\mathrm{for}\;\iota =1,2,$$

and

(51) $$\lim_{\varepsilon_{1}\to 0}\lim_{\varepsilon_{2}\to 0}\lim_{\epsilon \to 0}R_{\iota ,2}^{\varepsilon ,\epsilon }=0,\;\mathrm{in}\;L^{1}\left(\Omega ;L^{1}\left(0,T;L_{loc}^{1}\left(R^{d+1}\right)\right)\right),\;\mathrm{for}\;\iota =1,2.$$

On the other hand, for fixed $\varepsilon_{1}$, we have

$$R_{\iota ,3}^{\varepsilon ,\epsilon }=\sum_{i=1}^{d}\{\partial_{x_{i}}u_{\iota }^{\varepsilon ,\epsilon }\circ {\dot{M}}_{i}\left(t,v\right)-{\left[\partial_{x_{i}}u_{\iota }\circ {\dot{M}}_{i}\left(t,v\right)\right]}^{\varepsilon ,\epsilon }\}:=I_{\iota ,3}^{\varepsilon ,\epsilon }-\frac{1}{2}J_{\iota ,3}^{\varepsilon ,\epsilon },$$

where

$$\begin{array}{c} I_{\iota ,3}^{\varepsilon ,\epsilon }=\sum_{i=1}^{d}\{\partial_{x_{i}}u_{\iota }^{\varepsilon ,\epsilon }{\dot{M}}_{i}\left(t,v\right)-{\left[\partial_{x_{i}}u_{\iota }^{\varepsilon_{1}}{\dot{M}}_{i}\left(t,v\right)\right]}^{\varepsilon_{2},\epsilon }\},\\ J_{\iota ,3}^{\varepsilon ,\epsilon }=\sum_{i=1}^{d}\sum_{j=1}^{d}\sum_{k=1}^{n}\{\partial_{x_{i},x_{j}}^{2}u_{\iota }^{\varepsilon ,\epsilon }\sigma_{i,k}\left(t,v\right)\sigma_{j,k}\left(t,v\right)-{\left[\partial_{x_{i},x_{j}}^{2}u_{\iota }^{\varepsilon_{1}}\sigma_{i,k}\sigma_{j,k}\right]}^{\varepsilon_{2},\epsilon }\}. \end{array}$$

By Lemma 4 and (5),

(52) $$\lim_{\varepsilon_{2}\to 0}\lim_{\epsilon \to 0}I_{\iota ,3}^{\varepsilon ,\epsilon }=0,\;\mathrm{in}\;L^{2}\left(\Omega ;L^{2}\left(0,T;L_{loc}^{2}\left(R^{d+1}\right)\right)\right),\;\mathrm{for}\;\iota =1,2,$$

and by virtue of Lemma 3

(53) $$\lim_{\varepsilon_{1}\to 0}\lim_{\varepsilon_{2}\to 0}\lim_{\epsilon \to 0}J_{\iota ,3}^{\varepsilon ,\epsilon }=0,\;\mathrm{in}\;L^{1}\left(\Omega ;L^{1}\left(0,T;L_{loc}^{1}\left(R^{d+1}\right)\right)\right),\;\mathrm{for}\;\iota =1,2.$$

For $k_{1}$ and $k_{2}$ ($k_{2}$ is big enough) be fixed, if one lets ϵ tend to zero first, $\varepsilon_{2}$ approach to zero next, $\varepsilon_{1}$ incline to zero last, with the aid of (47)–(53) and Lemma 1, from (45), it leads to

E∫Rd+1[|u1(t,x,v)|+|u2(t,x,v)|−2u1(t,x,v)u2(t,x,v)]ξk2(v)θk1(x)dxdv⩽∫Rd+1[|u0,1(x,v)|+|u0,2(x,v)|−2u0,1(x,v)u0,2(x,v)]ξk2(v)θk1(x)dxdv+E∫0t∫Rd+1[|u1(s,x,v)|+|u2(s,x,v)|−2u1(s,x,v)u2(s,x,v)]ξk2f(v)·∇xθk1(x)dxdvds+E∫0t∫Rd+1[|u1(s,x,v)|+|u2(s,x,v)|−2u1(s,x,v)u2(s,x,v)]∂v[ξk2A(s,x,v)]θk1dxdvds(54)+∑i=1d∑j=1dE∫0t∫Rd+1[|u1|+|u2|−2u1u2]∂xi,xj2θk1(x)ξk2(v)ai,j(s,v)dxdvds.

Observing that $u_{\iota }\left(t,x,v\right)=\chi_{\rho_{\iota }\left(t,x\right)}\left(v\right)=1_{\left(0,\rho_{\iota }\left(t,x\right)\right)}\left(v\right)-1_{\left(\rho_{\iota }\left(t,x\right),0\right)}\left(v\right)\left(\iota =1,2\right)$, and

$$\left\{ \begin{array}{cc} {\left|1_{\left(0,\rho_{1}\right)}\left(v\right)-1_{\left(0,\rho_{2}\right)}\left(v\right)\right|}^{2}, & \mathrm{if}\;\rho_{1}⩾0,\rho_{2}⩾0,\\ {\left|1_{\left(0,\rho_{1}\right)}\left(v\right)+1_{\left(\rho_{2},0\right)}\left(v\right)\right|}^{2}, & \mathrm{if}\;\rho_{1}⩾0,\rho_{2}<0,\\ {\left|1_{\left(\rho_{1},0\right)}\left(v\right)+1_{\left(0,\rho_{2}\right)}\left(v\right)\right|}^{2}, & \mathrm{if}\;\rho_{1}<0,\rho_{2}⩾0,\\ {\left|1_{\left(\rho_{2},0\right)}\left(v\right)-1_{\left(\rho_{1},0\right)}\left(v\right)\right|}^{2}, & \mathrm{if}\;\rho_{1}<0,\rho_{2}<0, \end{array}=\{\begin{array}{cc} |1_{\left(0,\rho_{1}\right)}\left(v\right)-1_{\left(0,\rho_{2}\right)}\left(v\right)|, & \mathrm{if}\;\rho_{1}⩾0,\rho_{2}⩾0,\\ |1_{\left(0,\rho_{1}\right)}\left(v\right)+1_{\left(\rho_{2},0\right)}\left(v\right)|, & \mathrm{if}\;\rho_{1}⩾0,\rho_{2}<0,\\ |1_{\left(\rho_{1},0\right)}\left(v\right)+1_{\left(0,\rho_{2}\right)}\left(v\right)|, & \mathrm{if}\;\rho_{1}<0,\rho_{2}⩾0,\\ |1_{\left(\rho_{2},0\right)}\left(v\right)-1_{\left(\rho_{1},0\right)}\left(v\right)|, & \mathrm{if}\;\rho_{1}<0,\rho_{2}<0, \end{array}\right.$$

we have ${\left|u_{1}-u_{2}\right|}^{2}=\left|u_{1}-u_{2}\right|$.

Since $u_{1}$ and $u_{2}$ are supported in [−K,K] for *v*, if one chooses $k_{2}>K$, it follows from (54) that

E∫Rd+1|u1(t,x,v)−u2(t,x,v)|ξk2(v)θk1(x)dxdv⩽∫Rd+1|u0,1(x,v)−u0,2(x,v)|ξk2(v)θk1(x)dxdv+E∫0t∫Rd+1|u1(s,x,v)−u2(s,x,v)|ξk2f(v)·∇xθk1(x)dxdvds+E∫0t∫Rd+1|u1(s,x,v)−u2(s,x,v)|∂v[ξk2A(s,x,v)]θk1dxdvds+∑i=1d∑j=1dE∫0t∫Rd+1|u1(s,x,v)−u2(s,x,v)|∂xi,xj2θk1(x)ξk2(v)ai,j(s,v)dxdvds⩽∫Rd+1|u0,1(x,v)−u0,2(x,v)|θk1(x)dxdv+E∫0t∫Rd+1|u1(s,x,v)−u2(s,x,v)|f(v)·∇xθk1(x)dxdvds+E∫0t∫Rd+1|u1(s,x,v)−u2(s,x,v)|[∂vA(s,x,v)]+θk1dxdvds(55)+∑i=1d∑j=1dE∫0t∫Rd+1|u1(s,x,v)−u2(s,x,v)|∂xi,xj2θk1(x)ai,j(s,v)dxdvds.

By taking $k_{1}$ to infinity, with the help of (17), (18), then

$$\begin{array}{cc} & E\int_{R^{d+1}}\left|u_{1}\left(t,x,v\right)-u_{2}\left(t,x,v\right)\right|dxdv\\ ⩽ & \int_{R^{d+1}}\left|u_{0,1}\left(x,v\right)-u_{0,2}\left(x,v\right)\right|dxdv\\ & +\int_{0}^{t}{∥{\left[\partial_{v}A\left(s,\cdot ,\cdot \right)\right]}_{+}∥}_{L^{\infty }\left(R^{d}\times \left(-K,K\right)\right)}E\int_{R^{d+1}}\left|u_{1}\left(s,x,v\right)-u_{2}\left(s,x,v\right)\right|dxdvds, \end{array}$$

where *K* is given by (46).

Therefore

E∫Rd|ρ1(t,x)−ρ2(t,x)|dx=E∫Rd+1|u1(t,x,v)−u2(t,x,v)|dxdv⩽∫Rd+1|u0,1(x,v)−u0,2(x,v)|dxdvexp(∫0t∥[∂vA(s,·,·)]+∥L∞(Rd×(−K,K))ds)(56)=∫Rd|ρ0,1(x)−ρ0,2(x)|dxexp(∫0t∥[∂vA(s,·,·)]+∥L∞(Rd×(−K,K))ds).

From (56), we complete the proof.

It remains to prove Corollary 1. Indeed, if one mimics the above calculations, then

$$\begin{array}{cc} & E\int_{R^{d+1}}\left[u_{1}\left(t,x,v\right)-u_{2}\left(t,x,v\right)\right]dxdv\\ = & \int_{R^{d+1}}\left[u_{0,1}\left(x,v\right)-u_{0,2}\left(x,v\right)\right]dxdv\\ & +E\int_{0}^{t}\int_{R^{d+1}}\left[u_{1}\left(s,x,v\right)-u_{2}\left(s,x,v\right)\right]\partial_{v}A\left(s,x,v\right)dxdvds. \end{array}$$

Observing that

$${\left[u_{1}\left(t,x,v\right)-u_{2}\left(t,x,v\right)\right]}_{-}=\frac{|u_{1}-u_{2}|-\left(u_{1}-u_{2}\right)}{2},$$

hence

$$\begin{array}{cc} & E\int_{R^{d+1}}{\left[u_{1}\left(t,x,v\right)-u_{2}\left(t,x,v\right)\right]}_{-}dxdv\\ = & \frac{1}{2}E\int_{R^{d+1}}\left|u_{1}\left(t,x,v\right)-u_{2}\left(t,x,v\right)\right|dxdv-\frac{1}{2}E\int_{R^{d+1}}\left[u_{1}\left(t,x,v\right)-u_{2}\left(t,x,v\right)\right]dxdv\\ ⩽ & \frac{1}{2}\int_{R^{d+1}}[\left|u_{0,1}\left(x,v\right)-u_{0,2}\left(x,v\right)\right|-u_{0,1}\left(x,v\right)+u_{0,2}\left(x,v\right)]dxdv\\ & +\frac{1}{2}E\int_{0}^{t}\int_{R^{d+1}}[\left|u_{1}\left(s,x,v\right)-u_{2}\left(s,x,v\right)\right|-u_{1}\left(s,x,v\right)+u_{2}\left(s,x,v\right)]\partial_{v}A\left(s,x,v\right)dxdvds\\ = & \int_{R^{d+1}}{\left[u_{0,1}\left(x,v\right)-u_{0,2}\left(x,v\right)\right]}_{-}dxdv\\ & +E\int_{0}^{t}\int_{R^{d+1}}{\left[u_{1}\left(s,x,v\right)-u_{2}\left(s,x,v\right)\right]}_{-}\partial_{v}A\left(s,x,v\right)dxdvds\\ ⩽ & \int_{R^{d+1}}{\left[u_{0,1}\left(x,v\right)-u_{0,2}\left(x,v\right)\right]}_{-}dxdv\\ & +E\int_{0}^{t}{∥{\left[\partial_{v}A\left(s,\cdot ,\cdot \right)\right]}_{+}∥}_{L^{\infty }\left(R^{d}\times \left(-K,K\right)\right)}\int_{R^{d+1}}{\left[u_{1}\left(s,x,v\right)-u_{2}\left(s,x,v\right)\right]}_{-}dxdvds. \end{array}$$

The Grönwall inequality applies, one concludes

$$\begin{array}{cc} & E\int_{R^{d}}{\left[\rho_{1}\left(t,x\right)-\rho_{2}\left(t,x\right)\right]}_{-}dx\\ ⩽ & \int_{R^{d+1}}{\left[u_{0,1}\left(x,v\right)-u_{0,2}\left(x,v\right)\right]}_{-}dxdv\exp(\int_{0}^{t}{∥{\left[\partial_{v}A\left(s,\cdot ,\cdot \right)\right]}_{+}∥}_{L^{\infty }\left(R^{d}\times \left(-K,K\right)\right)}ds)\\ = & \int_{R^{d}}{\left[\rho_{0,1}\left(x\right)-\rho_{0,2}\left(x\right)\right]}_{-}dx\exp(\int_{0}^{t}{∥{\left[\partial_{v}A\left(s,\cdot ,\cdot \right)\right]}_{+}∥}_{L^{\infty }\left(R^{d}\times \left(-K,K\right)\right)}ds)=0, \end{array}$$

which implies $\rho_{1}⩽\rho_{2},\;P-a.s..$

**Remark** **5.** *As a special case, one confirms the uniqueness of stochastic entropy solutions for*

$$\left\{ \begin{array}{c} d\rho \left(t,x\right)+{\mathrm{div}}_{x}\left(F\left(\rho \right)\right)dt+\sum_{i=1}^{d}\partial_{x_{i}}\rho \left(t,x\right)\circ dW_{i}\left(t\right)=0,\;x\in R^{d},\;t>0,\\ {\rho \left(t,x\right)|}_{t=0}=\rho_{0}\left(x\right)\in L^{1}\left(R^{d}\right)\cap L^{\infty }\left(R^{d}\right), \end{array}\right.$$

*when* $F\in W_{loc}^{1,\infty }\left(R;R^{d}\right)$ *. However, we can not give an affirm answer on the problem whether the weak solution is unique or not, when F is non-regular (such as* $F\in L^{\infty }\left(R;R^{d}\right)$ *).*

## 5. Proof of Theorem 3

The conclusion will be reached in three steps, and to make the expression simpler and clearer, we use $R_{x}^{d}\times R_{v}$ instead of $R^{d+1}$.

- **Step 1:**σ=0. Now (11), (12) become to
  (57) $$\left\{ \begin{array}{c} \partial_{t}u\left(t,x,v\right)+f\left(v\right)\cdot \nabla_{x}u\left(t,x,v\right)+A\left(t,v\right)\partial_{v}u\left(t,x,v\right)=\partial_{v}m,\;\left(x,v\right)\in R_{x}^{d}\times R_{v},\;t>0,\\ {u\left(t,x,v\right)|}_{t=0}=\chi_{\rho_{0}\left(x\right)}\left(v\right),\;\left(x,v\right)\in R_{x}^{d}\times R_{v}. \end{array}\right.$$

We begin with building the existence of weak solutions for (57) by using the Bhatnagar-Gross-Krook approximation, i.e., for ε>0, we regard (57) as the ε→0 limit of the integro-differential equation

(58) $$\left\{ \begin{array}{c} \partial_{t}u_{\varepsilon }\left(t,x,v\right)+f\left(v\right)\cdot \nabla_{x}u_{\varepsilon }+A\left(t,v\right)\partial_{v}u_{\varepsilon }=\frac{1}{\varepsilon }[\chi_{\rho_{\varepsilon }\left(t,x\right)}-u_{\varepsilon }],\;\left(x,v\right)\in R_{x}^{d}\times R_{v},\;t>0,\\ u_{\varepsilon }{\left(t,x,v\right)|}_{t=0}=\chi_{\rho_{0}\left(x\right)}\left(v\right),\;\left(x,v\right)\in R_{x}^{d}\times R_{v}, \end{array}\right.$$

where $\rho_{\varepsilon }\left(t,x\right)=\int_{R}u_{\varepsilon }\left(t,x,v\right)dv.$

- **Assertion 1:** (58) is well-posed in $L_{loc}^{\infty }\left(\left[0,\infty \right);L^{\infty }\left(R_{x}^{d}\times R_{v}\right)\right)\cap C\left(\left[0,\infty \right);L^{1}\left(R_{x}^{d}\times R_{v}\right)\right)$.

Clearly, (58)_1_ grants an equivalent presentation

$$\partial_{t}Z_{\varepsilon }+f\left(v\right)\cdot \nabla_{x}Z_{\varepsilon }+A\left(t,v\right)\partial_{v}Z_{\varepsilon }=\frac{1}{\varepsilon }e^{\frac{t}{\varepsilon }}\chi_{e^{-\frac{t}{\varepsilon }}{\tilde{\rho }}_{\varepsilon }}\left(v\right),$$

here

$$Z_{\varepsilon }\left(t,x,v\right)=e^{\frac{t}{\varepsilon }}u_{\varepsilon }\left(t,x,v\right),\;{\tilde{\rho }}_{\varepsilon }=\int_{R}Z_{\varepsilon }\left(t,x,v\right)dv.$$

Due to the assumptions $F\in C^{1}\left(R;R^{d}\right)$ and $A\in L_{loc}^{1}\left(\left[0,\infty \right);W^{1,\infty }\left(R\right)\right)$, there is a unique global solution to the ODE

(59) $$\frac{d}{dt}{\left(X\left(t,x,v\right),V\left(t,v\right)\right)}^{⊤}={\left(f\left(V\right),A\left(t,V\right)\right)}^{⊤},\;\mathrm{with}\;{\left(X\left(t,x,v\right),V\left(t,v\right)\right)}^{⊤}{|}_{t=0}={\left(x,v\right)}^{⊤},$$

for every $\left(x,v\right)\in R_{x}^{d}\times R_{v}$.

Therefore, along the direction (59),

$$Z_{\varepsilon }\left(t,X\left(t\right),V\left(t\right)\right)=\frac{1}{\varepsilon }\int_{0}^{t}e^{\frac{s}{\varepsilon }}\chi_{e^{-\frac{s}{\varepsilon }}{\tilde{\rho }}_{\varepsilon }\left(s,X\left(s,x,v\right)\right)}\left(V\left(s,v\right)\right)ds+\chi_{\rho_{0}\left(x\right)}\left(v\right),$$

i.e.,

$$u_{\varepsilon }\left(t,X\left(t\right),V\left(t\right)\right)=\frac{1}{\varepsilon }\int_{0}^{t}e^{\frac{s-t}{\varepsilon }}\chi_{\rho_{\varepsilon }\left(s,X\left(s,x,v\right)\right)}\left(V\left(s,v\right)\right)ds+e^{-\frac{t}{\varepsilon }}\chi_{\rho_{0}\left(x\right)}\left(v\right).$$

Define $J\left(t,V\right)=|\partial_{v}V\left(t,v\right)|$, thanks to Euler’s formula, then

(60) $$\exp\left(-\int_{0}^{t}{\left[\partial_{v}A\left(s,V\left(s\right)\right)\right]}_{-}ds\right)⩽J\left(t,V\right)⩽\exp\left(\int_{0}^{t}{\left[\partial_{v}A\left(s,V\left(s\right)\right)\right]}_{+}ds\right),$$

whence, the inverse of the mapping ${\left(x,v\right)}^{⊤}\mapsto {\left(X,V\right)}^{⊤}$ exists and it forms a flow of homeomorphic. We thus have

(61) $$u_{\varepsilon }\left(t,x,v\right)=\frac{1}{\varepsilon }\int_{0}^{t}e^{\frac{s-t}{\varepsilon }}\chi_{\rho_{\varepsilon }\left(s,X_{t,s}\left(x,v\right)\right)}\left(V_{t,s}\left(v\right)\right)ds+e^{-\frac{t}{\varepsilon }}\chi_{\rho_{0}\left(X_{t,0}\left(x,v\right)\right)}\left(V_{t,0}\left(v\right)\right),$$

where ${\left(X_{t,s}\left(x,v\right),V_{t,s}\left(v\right)\right)}^{⊤}={\left(X_{s,t}^{-1}\left(x,v\right),V_{s,t}^{-1}\left(v\right)\right)}^{⊤}$, i.e.,

$$\left\{ \begin{array}{c} \frac{d}{dt}{\left(X_{s,t}\left(x,v\right),V_{s,t}\left(v\right)\right)}^{⊤}={\left(f\left(V_{s,t}\right),A\left(t,V_{s,t}\right)\right)}^{⊤},\;t⩾s,\\ {\left(X_{s,t}\left(x,v\right),V_{s,t}\left(v\right)\right)}^{⊤}{|}_{t=s}={\left(X\left(s,x,v\right),V\left(s,v\right)\right)}^{⊤}, \end{array}\right.$$

and ${\left(X_{s,t}\left(x,v\right),V_{s,s}\left(v\right)\right)}^{⊤}={\left(X\left(t,X\left(s,x,v\right),V\left(s,v\right)\right),V\left(t,V\left(s,v\right)\right)\right)}^{⊤}$.

For every $u\in L_{loc}^{\infty }\left(\left[0,\infty \right);L^{\infty }\left(R_{x}^{d}\times R_{v}\right)\right)\cap C\left(\left[0,\infty \right);L^{1}\left(R_{x}^{d}\times R_{v}\right)\right)$, we define a mapping $S_{\varepsilon }$ by:

(62) $$\left(S_{\varepsilon }u\right)\left(t,x,v\right)=\frac{1}{\varepsilon }\int_{0}^{t}e^{\frac{s-t}{\varepsilon }}\chi_{\rho^{u}\left(s,X_{t,s}\left(x,v\right)\right)}\left(V_{t,s}\left(v\right)\right)ds+e^{-\frac{t}{\varepsilon }}\chi_{\rho_{0}^{u}\left(X_{t,0}\left(x,v\right)\right)}\left(V_{t,0}\left(v\right)\right),$$

here

$$\rho^{u}\left(t,x\right)=\int_{R}u\left(t,x,v\right)dv,\;\rho_{0}^{u}\left(x\right)=\int_{R}u\left(0,x,v\right)dv=\rho_{0}\left(x\right).$$

We claim that $S_{\varepsilon }$ is well-defined in $L_{loc}^{\infty }\left(\left[0,\infty \right);L^{\infty }\left(R_{x}^{d}\times R_{v}\right)\right)\cap C\left(\left[0,\infty \right);L^{1}\left(R_{x}^{d}\times R_{v}\right)\right)$ and locally (in time) contractive in $C(\left[0,\infty \right);L^{1}\left(R_{x}^{d}\times R_{v}\right))$.

Initially, we collate that (62) is well-defined. Indeed,

(63) $${∥S_{\varepsilon }u∥}_{L^{\infty }\left(\left[0,T\right]\times R_{x}^{d}\times R_{v}\right)}⩽1,$$

and for every 0<T<∞,

sup0⩽t⩽T|1ε∫0tes−tεds∫Rxd×Rvχρu(s,Xt,s(x,v))(Vt,s(v))dxdv+e−tε∫Rxd×Rvχρ0u(Xt,0(x,v))(Vt,0(v))dxdv|=sup0⩽t⩽T|1ε∫0tes−tεds∫Rxd×Rvχρu(s,x)(v)exp(∫st∂vA(r,Vs,r(v))dr)dxdv+ e−tε∫Rxd×Rvχρ0u(x)(v)exp(∫0t∂vA(r,V0,r(v))dr)dxdv|(64)⩽exp(∫0T∥[∂vA]+∥L∞(R)(t)dt)[(1−e−Tε)∥u∥C([0,T];L1(Rxd×Rv))+∥ρ0u∥L1(Rxd×Rv)],

thus (62) is meaningful.

For every $g_{1},g_{2}\in L_{loc}^{\infty }\left(\left[0,\infty \right);L^{\infty }\left(R_{x}^{d}\times R_{v}\right)\right)\cap C\left(\left[0,\infty \right);L^{1}\left(R_{x}^{d}\times R_{v}\right)\right)$, an analogue calculation of (64) also leads to

∥Sεg1−Sεg2∥C([0,T];L1(Rxd×Rv))⩽sup0⩽t⩽T|1ε∫0tes−tεds∫Rxd×Rv|χρg1(s,Xt,s(x,v))(Vt,s(v))−χρg2(s,Xt,s(x,v))(Vt,s(v))|dxdv+ e−tε∫Rxd×Rv|χρ0g1(Xt,0(x,v))(Vt,0(v))−χρ0g2(Xt,0(x,v))(Vt,0(v))|dxdv|=sup0⩽t⩽T|1ε∫0tes−tεds∫Rxd×Rv|χρg1(s,x)(v)−χρg2(s,x)(v)|exp(∫st∂vA(r,Vs,r)dr)dxdv+ e−tε∫Rxd×Rv|χρ0g1(x)(v)−χρ0g2(x)(v)|exp(∫0t∂vA(r,V0,r(v))dr)dxdv|⩽exp(∫0T∥[∂vA]+∥L∞(R)dt)[(1−e−Tε)∥g1−g2∥C([0,T];L1(Rxd×Rv))(65)+∥g1,0−g2,0∥L1(Rxd×Rv)],

where $g_{1,0}=g_{1}\left(t=0\right)$ and $g_{2,0}=g_{2}\left(t=0\right)$.

In particular, if $g_{1,0}=g_{,0}=\chi_{\rho_{0}}$, from (65), for every T>0

$$\begin{array}{cc} & {∥S_{\varepsilon }g_{1}-S_{\varepsilon }g_{2}∥}_{C(\left[0,T\right];L^{1}\left(R_{x}^{d}\times R_{v}\right))}\\ ⩽ & \exp(\int_{0}^{T}{∥{\left[\partial_{v}A\right]}_{+}∥}_{L^{\infty }\left(R\right)}\left(t\right)dt)\left(1-e^{-\frac{T}{\varepsilon }}\right){∥g_{1}-g_{2}∥}_{C(\left[0,T\right];L^{1}\left(R_{x}^{d}\times R_{v}\right))}. \end{array}$$

Given above T>0 we select $T_{1}>0$ so small that $\exp(\int_{0}^{T}{∥{\left[\partial_{v}A\right]}_{+}∥}_{L^{\infty }\left(R\right)}\left(t\right)dt)\left(1-e^{-\frac{T_{1}}{\varepsilon }}\right)<1$. Then we apply the Banach fixed point theorem to find a unique $u_{\varepsilon }\in C\left(\left[0,T_{1}\right];L^{1}\left(R_{x}^{d}\times R_{v}\right)\right)$ solving the Cauchy problem (58). By (63), $u_{\varepsilon }\in L^{\infty }\left(\left[0,T\right];L^{\infty }\left(R_{x}^{d}\times R_{v}\right)\right)$, so $u_{\varepsilon }\left(T_{1}\right)\in L^{1}\left(R_{x}^{d}\times R_{v}\right))\cap L^{\infty }\left(R_{x}^{d}\times R_{v}\right)$. We then repeat the argument above to extend our solution to the time interval $\left[T_{1},2T_{1}\right]$. Continuing, after finitely many steps we construct a solution existing on the interval (0,T) for any T>0. From this, we demonstrate that there exists a unique $u_{\varepsilon }\in C\left(\left[0,\infty \right);L^{1}\left(R_{x}^{d}\times R_{v}\right)\right)\cap L_{loc}^{\infty }\left(\left[0,\infty \right);L^{\infty }\left(R_{x}^{d}\times R_{v}\right)\right)$ solving the Cauchy problem (58).

- **Assertion 2: (Comparison principle)**. For every $\rho_{0},\tilde{\rho_{0}}\in L^{1}\left(R^{d}\right)\cap L^{\infty }\left(R^{d}\right)$, the allied solutions $u_{\varepsilon }$ and ${\tilde{u}}_{\varepsilon }$ of (58) satisfy
  ∥[uε(t)−u˜ε(t)]+∥L1(Rxd×Rv)⩽exp(∫0t∥[∂vA]+∥L∞(R)(s)ds)∥[χρ0−χρ˜0]+∥L1(Rxd×Rv)(66)=exp(∫0t∥[∂vA]+∥L∞(R)(s)ds)∥[ρ0−ρ˜0]+∥L1(Rd),
  (67) $${∥\rho_{\varepsilon }\left(t\right)-{\tilde{\rho }}_{\varepsilon }\left(t\right)∥}_{L^{1}\left(R^{d}\right)}⩽\exp(\int_{0}^{t}{∥{\left[\partial_{v}A\right]}_{+}∥}_{L^{\infty }\left(R\right)}\left(s\right)ds){∥\rho_{0}-{\tilde{\rho }}_{0}∥}_{L^{1}\left(R^{d}\right)},$$
  (68) $${∥\rho_{\varepsilon }\left(t\right)∥}_{L^{\infty }\left(R^{d}\right)}⩽\exp(\int_{0}^{t}{∥{\left[\partial_{v}A\right]}_{+}∥}_{L^{\infty }\left(R\right)}\left(s\right)ds){∥\rho_{0}∥}_{L^{\infty }\left(R^{d}\right)}.$$

Furthermore, if $\rho_{0}⩽{\tilde{\rho }}_{0}$, for almost all $\left(t,x,v\right)\in \left(0,\infty \right)\times R_{x}^{d}\times R_{v}$, and almost all $\left(t,x\right)\in \left(0,\infty \right)\times R^{d}$,

(69) $$u_{\varepsilon }\left(t,x,v\right)⩽{\tilde{u}}_{\varepsilon }\left(t,x,v\right),\;\rho_{\varepsilon }\left(t,x\right)⩽{\tilde{\rho }}_{\varepsilon }\left(t,x\right).$$

Equation (69) holds mutatis mutandis from (66) and $u\left(t,x,v\right)=\chi_{\rho (t,x)}\left(v\right)$, it is sufficient to show (66)–(68). Since the calculations for (67) and (68) are analogue of (66), we only show (66) here. Let $\lambda_{\varepsilon }={\left[u_{\varepsilon }-{\tilde{u}}_{\varepsilon }\right]}_{+}$, by an approximation argument, it leads to

(70) $$\partial_{t}\lambda_{\varepsilon }\left(t,x,v\right)+f\left(v\right)\cdot \nabla_{x}\lambda_{\varepsilon }+A\left(t,v\right)\partial_{v}\lambda_{\varepsilon }=\frac{1}{\varepsilon }[\chi_{\rho_{\varepsilon }\left(t,x\right)}-\chi_{{\tilde{\rho }}_{\varepsilon }\left(t,x\right)}-\left(u_{\varepsilon }-{\tilde{u}}_{\varepsilon }\right)]\mathrm{sign}\lambda_{\varepsilon },$$

in $\left(0,\infty \right)\times R_{x}^{d}\times R_{v}$, with the initial data

(71) $$\lambda_{\varepsilon }{|}_{t=0}={\left[\chi_{\rho_{0}\left(x\right)}\left(v\right)-\chi_{{\tilde{\rho }}_{0}\left(x\right)}\left(v\right)\right]}_{+}.$$

Obviously, we have the following facts:

(72) $$[\chi_{\rho_{\varepsilon }\left(t,x\right)}-\chi_{{\tilde{\rho }}_{\varepsilon }\left(t,x\right)}-\left(u_{\varepsilon }-{\tilde{u}}_{\varepsilon }\right)]\mathrm{sign}\lambda_{\varepsilon }=[\chi_{\rho_{\varepsilon }\left(t,x\right)}-\chi_{{\tilde{\rho }}_{\varepsilon }\left(t,x\right)}]\mathrm{sign}\lambda_{\varepsilon }-\lambda_{\varepsilon },$$

and

(73) $$\int_{R}[\chi_{\rho_{\varepsilon }\left(t,x\right)}\left(v\right)-\chi_{{\tilde{\rho }}_{\varepsilon }\left(t,x\right)}\left(v\right)]\mathrm{sign}\lambda_{\varepsilon }\left(t,x,v\right)dv⩽\int_{R}\lambda_{\varepsilon }\left(t,x,v\right)dv.$$

Indeed, when $\rho_{\varepsilon }⩽{\tilde{\rho }}_{\varepsilon }$, (73) is nature and reversely,

$$\begin{array}{ccc} \int_{R}[\chi_{\rho_{\varepsilon }\left(t,x\right)}\left(v\right)-\chi_{{\tilde{\rho }}_{\varepsilon }\left(t,x\right)}\left(v\right)]\mathrm{sign}\lambda_{\varepsilon }\left(t,x,v\right)dv & ⩽ & \int_{R}[\chi_{\rho_{\varepsilon }\left(t,x\right)}\left(v\right)-\chi_{{\tilde{\rho }}_{\varepsilon }\left(t,x\right)}\left(v\right)]dv\\ & = & \int_{R}\left[u_{\varepsilon }-{\tilde{u}}_{\varepsilon }\right]dv\\ & ⩽ & \int_{R}\lambda_{\varepsilon }\left(t,x,v\right)dv. \end{array}$$

By (72), (73), from (70) it follows that

$$\partial_{t}\int_{R}\lambda_{\varepsilon }\left(t,x,v\right)dv+\int_{R}f\left(v\right)\cdot \nabla_{x}\lambda_{\varepsilon }dv⩽\int_{R}\partial_{v}A\left(t,v\right)\lambda_{\varepsilon }dv⩽{∥{\left[\partial_{v}A\left(t\right)\right]}_{+}∥}_{L^{\infty }\left(R\right)}\int_{R}\lambda_{\varepsilon }dv,$$

which suggests that for every $\varphi \in D(R^{d})$,

$$\frac{d}{dt}\int_{R_{x}^{d}\times R_{v}}\lambda_{\varepsilon }\left(t,x,v\right)\varphi \left(x\right)dxdv⩽\int_{R_{x}^{d}\times R_{v}}f\left(v\right)\cdot \nabla_{x}\varphi \lambda_{\varepsilon }dxdv+{∥{\left[\partial_{v}A\left(t\right)\right]}_{+}∥}_{L^{\infty }\left(R\right)}\int_{R_{x}^{d}\times R_{v}}\lambda_{\varepsilon }\varphi dxdv.$$

For every k∈N, we can choose φ such that for every 0⩽|x|⩽k, φ(x)=1, then by letting *k* tend to infinity, one deduces

(74) $$\frac{d}{dt}\int_{R_{x}^{d}\times R_{v}}\lambda_{\varepsilon }\left(t,x,v\right)dxdv⩽{∥{\left[\partial_{v}A\left(t\right)\right]}_{+}∥}_{L^{\infty }\left(R\right)}\int_{R_{x}^{d}\times R_{v}}\lambda_{\varepsilon }\left(t,x,v\right)dxdv.$$

On account of the fact: for every $\alpha_{1},\alpha_{2}\in R$,

(75) $$\int_{R}{\left[\chi_{\alpha_{1}}\left(v\right)-\chi_{\alpha_{2}}\left(v\right)\right]}_{+}dv={\left[\alpha_{1}-\alpha_{2}\right]}_{+},$$

from (74), by (71) and a Grönwall type argument, one arrives at (66).

- **Assertion 3:** With locally uniform convergence topology, $\left\{u_{\varepsilon }\right\}$ is pre-compact in $C(\left[0,\infty \right);L^{1}\left(R_{x}^{d}\times R_{v}\right))$ and $\left\{\rho_{\varepsilon }\right\}$ is pre-compact in $C(\left[0,\infty \right);L^{1}\left(R^{d}\right))$.

From (66) (with a slight change), we have for every $\left(\tilde{x},\tilde{v}\right)\in R_{x}^{d}\times R_{v}$, t∈(0,∞),

$$\begin{array}{cc} & {∥u_{\varepsilon }\left(t,\tilde{x}+\cdot ,\tilde{v}+\cdot \right)-u_{\varepsilon }\left(t,\cdot ,\cdot \right)∥}_{L^{1}\left(R_{x}^{d}\times R_{v}\right))}\\ ⩽ & \frac{1}{\varepsilon }\int_{0}^{t}e^{\frac{s-t}{\varepsilon }}{∥u_{\varepsilon }\left(s,\tilde{x}+\cdot ,\tilde{v}+\cdot \right)-u_{\varepsilon }\left(s,\cdot ,\cdot \right)∥}_{L^{1}\left(R_{x}^{d}\times R_{v}\right)}\exp(\int_{s}^{t}{∥{\left[\partial_{v}A\right]}_{+}∥}_{L^{\infty }\left(R\right)}\left(r\right)dr)ds\\ & +e^{-\frac{t}{\varepsilon }}\int_{R_{x}^{d}\times R_{v}}\left|\chi_{\rho_{0}\left(x+\tilde{x}\right)}\left(v+\tilde{v}\right)-\chi_{\rho_{0}\left(x\right)}\left(v\right)\right|dxdv\exp(\int_{0}^{t}{∥{\left[\partial_{v}A\right]}_{+}∥}_{L^{\infty }\left(R\right)}\left(r\right)dr). \end{array}$$

Thus

$$\begin{array}{cc} & {∥u_{\varepsilon }\left(t,\tilde{x}+\cdot ,\tilde{v}+\cdot \right)-u_{\varepsilon }\left(t,\cdot ,\cdot \right)∥}_{L^{1}\left(R_{x}^{d}\times R_{v}\right)}\\ ⩽ & \int_{R_{x}^{d}\times R_{v}}|\chi_{\rho_{0}\left(x+\tilde{x}\right)}\left(v+\tilde{v}\right)-\chi_{\rho_{0}\left(x\right)}\left(v\right)|dxdv\exp(\int_{0}^{t}{∥{\left[\partial_{v}A\right]}_{+}∥}_{L^{\infty }\left(R\right)}\left(s\right)ds). \end{array}$$

With the aid of (75), then for $\tilde{v}=0$, it follows that

$$\begin{array}{cc} & {∥\rho_{\varepsilon }\left(t,\tilde{x}+\cdot \right)-\rho_{\varepsilon }\left(t,\cdot \right)∥}_{L^{1}\left(R^{d}\right)}\\ = & \int_{R_{x}^{d}}|\int_{R_{v}}u_{\varepsilon }\left(t,\tilde{x}+x,v\right)dv-\int_{R}u_{\varepsilon }\left(t,x,v\right)dv|dx\\ ⩽ & \int_{R_{x}^{d}\times R_{v}}\left|u_{\varepsilon }\left(t,\tilde{x}+x,v\right)-u_{\varepsilon }\left(t,x,v\right)\right|dxdv\\ ⩽ & \int_{R_{x}^{d}\times R_{v}}|\chi_{\rho_{0}\left(x+\tilde{x}\right)}\left(v\right)-\chi_{\rho_{0}\left(x\right)}\left(v\right)|dxdv\exp(\int_{0}^{t}{∥{\left[\partial_{v}A\right]}_{+}∥}_{L^{\infty }\left(R\right)}\left(r\right)dr), \end{array}$$

which implies for every 0<T<∞, $\left\{u_{\varepsilon }\right\}$ is contained in a compact set of $C(\left[0,T\right];L_{loc}^{1}\left(R_{x}^{d}\times R_{v}\right))$, $\left\{\rho_{\varepsilon }\right\}$ is pre-compact in $C(\left[0,T\right];L_{loc}^{1}\left(R^{d}\right))$. Hence by appealing to the Arzela-Ascoli theorem, with any sequence $\left\{\varepsilon_{k}\right\}$, $\varepsilon_{k}\to 0$ as k→∞, is associated two subsequences (for ease of notation, we also denote them by themselves) $\left\{u_{\varepsilon_{k}}\right\}$ and $\left\{\rho_{\varepsilon_{k}}\right\}$, such that

$$u_{\varepsilon_{k}}⟶u\in C\left(\left[0,T\right];L_{loc}^{1}\left(R_{x}^{d}\times R_{v}\right)\right),\;\rho_{\varepsilon_{k}}⟶\rho \in C\left(\left[0,T\right];L_{loc}^{1}\left(R^{d}\right)\right),\;\mathrm{as}\;k\to \infty .$$

On the other hand, by (63) and the lower semi-continuity,

$$\begin{array}{c} u\in L_{loc}^{\infty }\left(\left[0,\infty \right);L^{\infty }\left(R_{x}^{d}\times R_{v}\right)\right)\cap C\left(\left[0,\infty \right);L^{1}\left(R_{x}^{d}\times R_{v}\right)\right),\\ \rho \in L_{loc}^{\infty }\left(\left[0,\infty \right);L^{\infty }\left(R^{d}\right)\right)\cap C\left(\left[0,\infty \right);L^{1}\left(R^{d}\right)\right). \end{array}$$

- **Assertion 4:**$\frac{1}{\varepsilon }[\chi_{\rho_{\varepsilon }}-u_{\varepsilon }]=\partial_{v}m_{\varepsilon }$, where $m_{\varepsilon }⩾0$ is continuous in *t* and bounded uniformly in ε.

Let $\left(t,x\right)\in \left(0,\infty \right)\times R^{d}$ be fixed, assuming without loss of generality that $\rho_{\varepsilon }⩾0$, define

$$m_{\varepsilon }\left(t,x,v\right)=\frac{1}{\varepsilon }\int_{-\infty }^{v}[\chi_{\rho_{\varepsilon }\left(t,x\right)}\left(r\right)-u_{\varepsilon }\left(t,x,r\right)]dr.$$

In view of (61),

$$u_{\varepsilon }\left(t,x,r\right)\in \{\begin{array}{cc} {}[0,1], & \mathrm{when}\;r>0,\\ {}[-1,0], & \mathrm{when}\;r<0. \end{array}$$

Hence $m_{\varepsilon }\left(t,x,v\right)$ is non-decreasing on $\left(-\infty ,\rho_{\varepsilon }\right)$ and non-increasing on $\left[\rho_{\varepsilon },\infty \right)$. On the other hand, $m_{\varepsilon }\left(t,x,-\infty \right)=m_{\varepsilon }\left(t,x,\infty \right)=0$, we conclude $m_{\varepsilon }⩾0$.

Since $\rho_{0}\in L^{1}\left(R^{d}\right)\cap L^{\infty }\left(R^{d}\right)$, owing to (60), (61) and (68), and the condition $A\in L_{loc}^{1}\left(\left[0,\infty \right);W^{1,\infty }\left(R\right)\right)$, then

$$\mathrm{supp}m_{\varepsilon }\subset \left[0,T\right]\times R_{x}^{d}\times \left[-K,K\right],$$

where $K={∥\rho ∥}_{L^{\infty }\left(\left(0,T\right)\times R^{d}\right)}\exp(\int_{0}^{T}{∥\partial_{v}A\left(t\right)∥}_{L^{\infty }\left(R\right)}ds)$.

For the above fixed T>0,

$$\begin{array}{cc} & \int_{0}^{T}dt\int_{R^{d}}dx\int_{R}m_{\varepsilon }\left(t,x,v\right)dv\\ = & \int_{0}^{T}dt\int_{R^{d}}dx\int_{-K}^{K}dv\int_{-K}^{v}\left[\partial_{t}u_{\varepsilon }+f\left(r\right)\cdot \nabla_{x}u_{\varepsilon }+A\left(t,r\right)\partial_{r}u_{\varepsilon }\right]dr\\ = & \int_{0}^{T}dt\int_{R^{d}}dx\int_{-K}^{K}dv\int_{-K}^{v}\left[\partial_{t}u_{\varepsilon }\left(t,x,r\right)+A\left(t,r\right)\partial_{r}u_{\varepsilon }\right]dr\\ ⩽ & 2K[{∥u_{\varepsilon }\left(T\right)∥}_{L^{1}\left(R_{x}^{d}\times R_{v}\right)}+{∥u_{\varepsilon }\left(0\right)∥}_{L^{1}\left(R_{x}^{d}\times R_{v}\right)}]+\int_{0}^{T}dt\int_{R^{d}}dx\int_{-K}^{K}A\left(t,v\right)u_{\varepsilon }\left(t,x,v\right)dv\\ & -\int_{0}^{T}dt\int_{R^{d}}dx\int_{-K}^{K}dv\int_{-K}^{v}\partial_{r}A\left(t,r\right)u_{\varepsilon }\left(t,x,r\right)dr. \end{array}$$

Combining (68), we arrive at

$$\begin{array}{cc} & \int_{0}^{T}dt\int_{R^{d}}dx\int_{R}m_{\varepsilon }\left(t,x,v\right)dv\\ ⩽ & 4K^{2}{∥\rho_{0}∥}_{L^{1}\left(R^{d}\right)}+\left(1+2K\right)\int_{0}^{T}dt\int_{R^{d}}dx\int_{-K}^{K}{∥\partial_{v}A\left(t\right)∥}_{W^{1,\infty }\left(R\right)}\left|u_{\varepsilon }\left(t,x,v\right)\right|dv. \end{array}$$

Whence $m_{\varepsilon }$ is bounded uniformly in ε.

By extracting a unlabeled subsequence, one achieves

$$m_{\varepsilon }\to m⩾0\;\mathrm{in}\;D^{\prime }\left(\left[0,\infty \right)\times R_{x}^{d}\times R_{v}\right).$$

In order to show that *m* yields the properties stated in Theorem 1, it suffices to check that it is continuous in *t*, and by a translation, it remains to demonstrate the continuity at zero. But this fact is obvious, so the required result is complete.

- **Assertion 5:**$u\left(t,x,v\right)=\chi_{\rho (t,x)}\left(v\right)$ and ρ solves (23), (2) with $M_{i}\equiv 0$ (1⩽i⩽d). In addition, for every $\rho_{0},\tilde{\rho_{0}}\in L^{1}\left(R^{d}\right)\cap L^{\infty }\left(R^{d}\right)$, the related solutions *u* and $\tilde{u}$ of (57) fulfill
  ∥[u(t)−u˜(t)]+∥L1(Rxd×Rv)⩽exp(∫0t∥[∂vA]+∥L∞(R)(s)ds)∥[χρ0−χρ˜0]+∥L1(Rxd×Rv)(76)=exp(∫0t∥[∂vA]+∥L∞(R)(s)ds)∥[ρ0−ρ˜0]+∥L1(Rd),
  (77) $${∥\rho \left(t\right)-\tilde{\rho }\left(t\right)∥}_{L^{1}(R^{d})}⩽\exp(\int_{0}^{t}{∥{\left[\partial_{v}A\right]}_{+}∥}_{L^{\infty }\left(R\right)}\left(s\right)ds){∥\rho_{0}-{\tilde{\rho }}_{0}∥}_{L^{1}\left(R^{d}\right)},$$
  (78) $${∥\rho \left(t\right)∥}_{L^{\infty }\left(R^{d}\right)}⩽\exp(\int_{0}^{t}{∥{\left[\partial_{v}A\right]}_{+}∥}_{L^{\infty }\left(R\right)}\left(s\right)ds){∥\rho_{0}∥}_{L^{\infty }\left(R^{d}\right)}.$$

Furthermore, if $\rho_{0}⩽{\tilde{\rho }}_{0}$, for almost all $\left(t,x,v\right)\in \left(0,\infty \right)\times R_{x}^{d}\times R_{v}$, and almost all $\left(t,x\right)\in \left(0,\infty \right)\times R^{d}$,

(79) $$u\left(t,x,v\right)⩽\tilde{u}\left(t,x,v\right),\;\rho \left(t,x\right)⩽\tilde{\rho }\left(t,x\right).$$

In particular, if $\rho_{0}⩾0$, then u⩾0, ρ⩾0.

Observing that $u_{\varepsilon }\to u$, $\rho_{\varepsilon }\to \rho$ and $m_{\varepsilon }\to m$, so $u_{\varepsilon }\left(t,x,v\right)-\chi_{\rho_{\varepsilon }}\left(v\right)\to 0$ and then $u=\chi_{\rho (t,x)}\left(v\right)$. Moreover, ρ is a weak solution of (57).

With the help of (66)–(69), the rest of the assertion is clear.

**Step 2:** Existence of stochastic weak solutions to the Cauchy problem:

(80) $$\left\{ \begin{array}{c} \partial_{t}u+f\left(v\right)\cdot \nabla_{x}u+\sum_{i=1}^{d}\partial_{x_{i}}u\circ {\dot{M}}_{i}\left(t\right)+A\left(t,v\right)\partial_{v}u=\partial_{v}m,\;\left(x,v\right)\in R_{x}^{d}\times R_{v},\;t>0,\\ {u\left(t,x,v\right)|}_{t=0}=\chi_{\rho_{0}}\left(v\right),\;\left(x,v\right)\in R_{x}^{d}\times R_{v}. \end{array}\right.$$

Before handling the general σ, we review some notions. For any $a\in R^{d}$, set $\tau_{a}$ by

$$\tau_{a}\varphi \left(x\right)=\varphi \left(x+a\right),\;\mathrm{for}\;\mathrm{every}\;\varphi \in C\left(R^{d}\right),$$

and the pullback mapping of *m* by $\tau_{a}^{*}$ is defined by

$$\tau_{a}^{*}m\left(\tilde{\phi }\right)=m\left(\tau_{-a}\tilde{\phi }\right)=\int_{0}^{\infty }dt\int_{R^{d}}dx\int_{R}\tilde{\phi }\left(t,x-a,v\right)dv,$$

for every $\tilde{\phi }\in D\left(\left[0,\infty \right)\times R_{x}^{d}\times R_{v}\right).$

Let us consider the Cauchy problem below

(81) $$\left\{ \begin{array}{c} \partial_{t}\tilde{u}\left(t,x,v\right)+f\left(v\right)\cdot \nabla_{x}\tilde{u}+A\left(t,v\right)\partial_{v}\tilde{u}\left(t,x,v\right)=\tau_{M(t)}^{*}\partial_{v}m,\;\left(x,v\right)\in R_{x}^{d}\times R_{v},\;t>0,\\ \tilde{u}{\left(t,x,v\right)|}_{t=0}=\chi_{\rho_{0}\left(x\right)}\left(v\right),\;\left(x,v\right)\in R_{x}^{d}\times R_{v}. \end{array}\right.$$

The arguments employed in (57) for $\partial_{v}m$ adapted to $\tau_{M(t)}^{*}\partial_{v}m=\partial_{v}\tau_{M(t)}^{*}m$ in (81) now, produces that there is a $\tilde{u}\left(\omega \right)\in L_{loc}^{\infty }\left(\left[0,\infty \right);L^{\infty }\left(R_{x}^{d}\times R_{v}\right)\right)\cap C\left(\left[0,\infty \right);L^{1}\left(R_{x}^{d}\times R_{v}\right)\right)$ solving (81). Note that $\tau_{M(t)}^{*}\partial_{v}m$ is $F_{t}$-adapted with values in $D^{\prime }\left(R_{x}^{d}\times R_{v}\right)$, thus for every $\phi \in D(R_{x}^{d}\times R_{v})$, $\int_{R_{x}^{d}\times R_{v}}\tilde{u}\left(t,x,v\right)\phi \left(x,v\right)dxdv$ is $F_{t}$-adapted. Besides, by Assertion 5, $\tilde{u}\in L^{\infty }\left(\Omega ;L_{loc}^{\infty }\left(\left[0,\infty \right);L^{\infty }\left(R_{x}^{d}\times R_{v}\right)\right)\right)\cap C\left(\left[0,\infty \right);L^{1}\left(R_{x}^{d}\times R_{v}\times \Omega \right)\right)$.

Hence, upon using Itô-Wentzell’s formula (see [38]) to $G\left(y\right)=\int_{R_{x}^{d}\times R_{v}}\tilde{u}\left(t,x,v\right)\phi \left(x+y,v\right)dxdv$, one gains

$$\begin{array}{cc} & \int_{R_{x}^{d}\times R_{v}}\tilde{u}\left(t,x,v\right)\phi \left(x+M_{t},v\right)dxdv-\int_{R_{x}^{d}\times R_{v}}\chi_{\rho_{0}\left(x\right)}\left(v\right)\phi \left(x,v\right)dxdv\\ = & \int_{0}^{t}ds\int_{R_{x}^{d}\times R_{v}}\tilde{u}f\left(v\right)\cdot \nabla_{x}\phi \left(x+M_{s},v\right)dxdv+\int_{0}^{t}ds\int_{R_{x}^{d}\times R_{v}}\tilde{u}\partial_{v}\left[A\left(s,v\right)\phi \left(x+M_{s},v\right)\right]dxdv\\ & +\sum_{i=1}^{d}\int_{0}^{t}M_{i}\left(\circ ds\right)\int_{R_{x}^{d}\times R_{v}}\tilde{u}\left(s,x,v\right)\partial_{x_{i}}\phi \left(x+M_{s},v\right)dxdv-\int_{0}^{t}\int_{R_{x}^{d}\times R_{v}}\partial_{v}\phi \left(x,v\right)m\left(ds,dx,dv\right). \end{array}$$

Let $u\left(t,x,v\right)=\tilde{u}\left(t,x-M_{t},v\right)$, then $\tilde{u}\in L^{\infty }\left(\Omega ;L_{loc}^{\infty }\left(\left[0,\infty \right);L^{\infty }\left(R_{x}^{d}\times R_{v}\right)\right)\right)\cap C\left(\left[0,\infty \right);L^{1}\left(R_{x}^{d}\times R_{v}\times \Omega \right)\right)$, which is $F_{t}$-adapted, and

(82) $$\begin{array}{cc} & \int_{R_{x}^{d}\times R_{v}}u\left(t,x,v\right)\phi \left(x,v\right)dxdv-\int_{R_{x}^{d}\times R_{v}}\chi_{\rho_{0}\left(x\right)}\left(v\right)\phi \left(x,v\right)dxdv\\ = & \int_{0}^{t}ds\int_{R_{x}^{d}\times R_{v}}u\left(s,x,v\right)f\left(v\right)\cdot \nabla_{x}\phi \left(x,v\right)dxdv+\int_{0}^{t}ds\int_{R_{x}^{d}\times R_{v}}u\left(s,x,v\right)\partial_{v}\left[A\left(s,v\right)\phi \left(x,v\right)\right]dxdv\\ & +\sum_{i=1}^{d}\int_{0}^{t}M_{i}\left(\circ ds\right)\int_{R_{x}^{d}\times R_{v}}u\left(s,x,v\right)\partial_{x_{i}}\phi \left(x,v\right)dxdv-\int_{0}^{t}\int_{R_{x}^{d}\times R_{v}}\partial_{v}\phi \left(x,v\right)m\left(ds,dx,dv\right). \end{array}$$

Thanks to (82) and Remark 3, hence there exists a stochastic weak solution to (80).

**Step 3:** Existence of stochastic entropy solutions to (23), (2).

Due to Step 2, one claims that

$$u\left(t,x,v\right)=\chi_{\rho (t,x)}\left(v\right)\;\mathrm{and}\;\rho \in L^{\infty }\left(\Omega ;L_{loc}^{\infty }\left(\left[0,\infty \right);L^{\infty }\left(R^{d}\right)\right)\right)\cap C\left(\left[0,\infty \right);L^{1}\left(R^{d}\times \Omega \right)\right).$$

Theorem 1 (ii) applies, ρ is a stochastic entropy solution of (23), (2).

**Remark** **6.** *When* A(t,ρ)=ξ(t)ρ(t,x) *, then analogue calculations of (77), (78) also yield that*

$${∥\rho \left(t\right)∥}_{L^{\iota }\left(R^{d}\right)}⩽\exp(\int_{0}^{t}\xi \left(s\right)ds){∥\rho_{0}∥}_{L^{\iota }\left(R^{d}\right)},\;for\;t\in \left[0,\infty \right)\;and\;\iota =1\;or\;\infty .$$

*Whence for every* p∈[1,∞] *,*

(83) $${∥\rho \left(t\right)∥}_{L^{p}\left(R^{d}\right)}⩽C\exp\left(\int_{0}^{t}\xi \left(s\right)ds\right).$$

*If there is a positive real number* c>0 *such that* ξ⩽−c *, then with probability one, the unique stochastic entropy solution ρ is exponentially stable. If for some real number* $\alpha_{1},r_{1}>0$ *, ξ possesses the below form*

$$\xi \left(t\right)=\{\begin{array}{c} -\frac{\alpha_{1}}{t},\;when\;t\in \left(r_{1},\infty \right),\\ \xi_{1}\left(t\right),\;when\;t\in \left[0,r_{1}\right], \end{array}$$

*where* $\xi_{1}\in L^{1}\left(\left[0,r_{1}\right]\right)$ *, then from (83),*

$${∥\rho \left(t\right)∥}_{L^{p}\left(R^{d}\right)}⩽\{\begin{array}{cc} C, & t\in [0,r_{1}],\\ \frac{C}{t^{\alpha_{1}}}, & t\in \left[r_{1},\infty \right), \end{array}$$

*which implies ρ is asymptotically stable.*

## 6. Conclusions

In recent years, people have made broad research about the uniqueness and existence of solutions for the conservation law

(84) $$\partial_{t}\rho \left(t,x\right)+{\mathrm{div}}_{x}\left(F\left(\rho \right)\right)=0,\;x\in R^{d},\;t>0,$$

with a stochastic perturbation. Most of these works are concentrated on the multiplicative type:

(85) $$d\rho \left(t,x\right)+{\mathrm{div}}_{x}\left(F\left(\rho \right)\right)dt=A\left(t,x,\rho \right)d\tilde{W}\left(t\right),\;x\in D,\;t>0,$$

where $\tilde{W}$ is a 1-dimensional Wiener process or a cylindrical Wiener process, $D\subset R^{d}$ is a bounded domain or $D=R^{d}$. However, for Equation (85), if we take the spatial average for ρ, then it satisfies

$$\int_{D}\rho \left(t,x\right)dx=\int_{D}\rho_{0}\left(x\right)dx+\int_{0}^{t}\int_{D}A\left(s,x,\rho \left(s,x\right)\right)dxd\tilde{W}\left(s\right).$$

It seems difficult to provide any bound on the average for the last term in the above identity. So the mass is not preserved in general. But if one considers the scalar conservation (84) with the noise given by $\sum_{i=1}^{d}\sum_{j=1}^{n}\partial_{x_{i}}B_{i,j}\left(t,\rho \right)\circ dW_{j}\left(t\right)$,

(86) $$d\rho \left(t,x\right)+{\mathrm{div}}_{x}\left(F\left(\rho \right)\right)dt+\sum_{i=1}^{d}\sum_{j=1}^{n}\partial_{x_{i}}B_{i,j}\left(t,\rho \right)\circ dW_{j}\left(t\right)=0,\;x\in D,\;t>0,$$

then

$$\int_{D}\rho \left(t,x\right)dx=\int_{D}\rho_{0}\left(x\right)dx.$$

Therefore, with such noise, the mass is preserved exactly. From the point of this view, the noise given here is more reasonable, and compared with the existing research works [5,6,7,8,9,10,11,12,13,14,15,16,17,18], this idea is new.

On the other hand, when we discuss the conservation law (84), $L^{\infty }$ is a natural space on which the solutions are well-posed. But if one perturbs the Equation (84) by the noise $A\left(t,x,\rho \right)d\tilde{W}\left(t\right)$, even the initial data is bounded, the solution is not bounded since the maximum principle is not available. Therefore, $L^{\infty }$ is not a natural space on which the solutions exist. Even though, if we assume further that *A* has compact support, then $L^{\infty }$ solutions will exist [5,6]. However, in the present paper, by using the stochastic kinetic formulation, we also found the existence for bounded solutions without the compact support assumptions on coefficients for stochastic balance law (1). Moreover, we prove the uniqueness for stochastic entropy solutions without any assumptions on the growth rates of the coefficients to (1). Compared with the known results, the existence and uniqueness for stochastic entropy solutions established in the present paper are new as well. 
